# Potential of SERS and proteomics for biomarker detection in cancer cells

**DOI:** 10.1007/s00216-026-06459-5

**Published:** 2026-03-27

**Authors:** David Lilek, Anna Mayr, Christoph Grossinger, Justyna Rechthaler, Lukas Steininger, Daniel Zimmermann, Sonja Gamsjaeger, Bodo D. Wilts, Maurizio Musso, Christoph Wiesner, Agnes Grünfelder, Birgit Herbinger, Katerina Prohaska

**Affiliations:** 1 Biotech Campus Tulln, University of Applied Sciences Wiener Neustadt, Konrad-Lorenz Straße 10, 3430 Tulln, Austria; 2 Department of Chemistry and Physics of Materials, Paris Lodron University Salzburg, Jakob-Haringer-Str. 2a, 5020 Salzburg, Austria; 3 Ludwig Boltzmann Institute of Osteology at the Hanusch Hospital of OEGK and AUVA Trauma Centre Meidling, 1st Medical Department, Hanusch Hospital, Heinrich Collin Str. 30, A-1140 Vienna, Austria; 4 Institute Biotechnology, IMC Krems University of Applied Sciences, Krems an der Donau, Austria

**Keywords:** Proteomics, SERS (surface-enhanced Raman spectroscopy), Machine learning, Multi-omics, Hodgkin lymphoma, Biomarker detection

## Abstract

This study investigates the use of quantitative LC-MS/MS-based proteomics and surface-enhanced Raman spectroscopy (SERS) for biomarker detection in classical Hodgkin lymphoma (HL). Two HL cell models with distinct TP53 status were utilized to evaluate the effects of etoposide, a DNA-damaging chemotherapeutic agent, and resveratrol, a polyphenolic compound with known chemosensitizing activity. For SERS, the best performance was achieved by applying logistic regression to classify different treatment conditions and identify discriminative spectral features in the data. Proteomics showed highly reproducible and accurate results with relative standard deviations of below 5% for the sample preparation and about 2% for the measurements. Proteomic profiling revealed a TP53-dependent organization of metabolic and stress-response pathways and demonstrated that cryopreserved aliquots yielded the most consistent proteomic signatures. Treatment-dependent regulation of key biomarker proteins showed direct correspondence to specific SERS features, such as reduced nucleotide/cytochrome-associated signals and enhanced amide and aromatic amino acid signals. Our findings highlight the strength of applying reproducible proteomic profiling with machine learning–guided SERS analysis to improve molecular interpretation and to validate potential biomarkers in cancer research. Future work will focus on refining the analytical workflow and extending it toward integrative multi-omics applications, enabling more comprehensive biomarker detection and mechanistic insight into classical Hodgkin lymphoma. This strategy will be extended to additional model systems—such as metastatic melanoma, melanocytes, and ultimately patient-derived leukemia cells—to help bridge the gap toward clinical translation.

**Graphical abstract:**

An analytical approach that enables reproducible and complementary biomarker discovery in Hodgkin lymphoma cells by applying robust SERS-based spectral classification with quantitative LC-MS/MS proteomics for molecular validation. It emphasizes reproducibility and data complementarity while identifying treatment-specific molecular signatures.

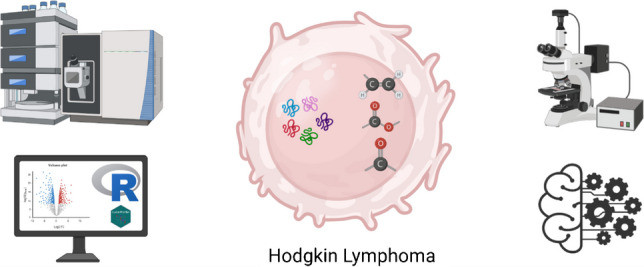

**Supplementary Information:**

The online version contains supplementary material available at 10.1007/s00216-026-06459-5.

## Introduction

Cancer remains one of the globally leading causes of death, and its incidence is projected to increase in the coming decades, particularly among populations with higher life expectancy, educational attainment, and standard of living [1]. Among the many types of cancer, lymphomas form a major category of hematologic malignancies. Hodgkin lymphoma (HL) accounts for approximately 2–2.5% of all cancer diagnoses in the USA and presents a clinically and biologically diverse disease entity. Therapeutically, HL is considered one of the most curable malignancies, with modern combined chemotherapy protocols [2], achieving cure rates of approximately 80% and survival rates approaching 95% [2]. Nonetheless, a subset of patients, particularly those with refractory or relapsed (R/R) disease, respond poorly to standard therapies. For these individuals, novel therapeutic strategies are urgently needed. Recent developments include the use of monoclonal antibodies, immune checkpoint inhibitors [3], and CD30-targeted agents [4]. Given the heterogeneity of HL and the clinical challenges posed by R/R cases, there is growing interest in identifying reliable biomarkers that can help stratify patients, predict treatment outcomes, and guide therapeutic decision-making [5]. Despite significant advances in molecular profiling technologies, validated biomarkers that can be routinely used in clinical practice remain scarce, emphasizing the need for robust experimental models and integrative analytical strategies. For this reason, experimental model systems such as HL-derived cancer cell lines play an essential role in advancing our understanding of disease biology and therapy resistance, thereby serving as a foundation for biomarker discovery, drug screening, and mechanistic investigations.

The emergence of omics technologies such as genomics, transcriptomics, proteomics, and metabolomics has fundamentally transformed the field of cancer research by enabling comprehensive molecular profiling of tumors [6]. These high-throughput approaches allow the simultaneous analysis of thousands of biomolecules [7], offering deep insights into molecular mechanisms underlying oncogenesis and tumor progression [6, 8]. Through the integration of different omics approaches, researchers can construct detailed molecular portraits of cancer, thereby supporting improved diagnosis, prognosis, risk stratification, and the development of personalized treatment strategies [5, 9]. Despite the recent advances, the field still faces considerable challenges: many studies are based on small or heterogeneous sample cohorts, often affected by missing data and limited reproducibility of reported biomarkers [5, 10, 11]. To date, only a small fraction of candidate biomarkers identified through omics research have progressed into routine diagnostic use [6, 8, 12]. This translational bottleneck underscores the need for prospective, well-controlled, and technically standardized studies to ensure clinical validity and utility of emerging biomarkers [6, 9].

Within the broader framework of cancer biomarker discovery, surface-enhanced Raman scattering (SERS) has emerged as a highly promising analytical technique that enables rapid, label-free, and non-destructive molecular characterization. SERS complements optical spectroscopic approaches whose integration into multi-omics workflows remains limited but holds great potential to bridge molecular and biophysical information. SERS enhances the inherently weak Raman signal by several orders of magnitude through plasmonic field amplification using metallic nanoparticles, thereby allowing the detection of subtle biochemical changes with high sensitivity [13–16]. Intensive research has demonstrated the suitability of SERS for detecting molecular alterations induced by chemotherapeutic agents and for identifying disease-specific biomarker signatures across various cancer types [13, 17–26]. Notably, several studies have applied SERS in the context of HL, confirming its potential for disease characterization and subclassification [27–29]. Despite these promising results, the broader adoption of SERS in biomedical research and clinical translation remains limited by challenges in reproducibility. Consequently, robust data analysis pipelines including quality control are essential to ensure reliable spectral interpretation and to mitigate technical noise [30–32]. While SERS enables rapid molecular fingerprinting, mass spectrometry (MS)–based proteomics provides a complementary strategy based on its ability to detect and quantify thousands of proteins in a high-throughput, sensitive, and quantitative manner [33, 34]. In contrast to genomic methods, which reflect static genetic information, proteomics reflects the dynamic functional state of the cell that is critical for understanding disease mechanisms and therapy response [33]. Despite ongoing optimization of the nano-LC-MS/MS method, sample preparation remains a key factor of reproducibility and sensitivity. This underscores the need for standardized and robust protocols for protein extraction, digestion, and fractionation, which are essential for reliable biomarker validation and future clinical translation [35–37]. This, combined with the unique pathobiology of Hodgkin lymphoma, offers opportunities for biomarker-based risk stratification and therapeutic innovation [3, 4].

From a data analysis perspective, integrating and interpreting multi-omics datasets, particularly when combining proteomic and spectroscopic data, poses substantial analytical challenges [5, 38–40]. Datasets often vary widely in size, structure, and quality, and are commonly affected by issues such as missing values, batch effects, and imbalance across experimental groups [33, 36, 41–43]. These limitations can introduce bias, hinder reproducibility, and compromise the biological and clinical validity of derived models. To address these issues, advanced computational strategies are required [8, 32, 44]. Despite the rich information content of proteomic data, machine learning remains underutilized in this field, applied in only a small fraction of studies, whereas SERS data analysis relies more heavily on supervised learning approaches [35, 36, 41, 42]. Across both domains, the quality and preprocessing of data, as well as appropriate dimensionality reduction and model validation, are decisive for obtaining biologically meaningful and clinically relevant results, as demonstrated in our previous studies [35, 45].

In this study, two classical Hodgkin lymphoma (HL) cell lines were used [46]: one with wildtype TP53 (L-428) and another carrying a TP53 mutation (L-540), which impairs DNA damage response and apoptotic signaling. Both cell lines were treated with etoposide and resveratrol. The aim of the study was to establish a controlled in vitro methodological framework, rather than to perform clinical validation, addressing the current lack of approaches capable of linking rapid phenotypic screening with molecular specificity. For this purpose, we combined the functional depth of LC-MS/MS-based bottom-up proteomics with the rapid, label-free fingerprinting capabilities of SERS. This analytical strategy enabled us to identify treatment-induced molecular alterations and potential biomarker candidates in HL. To ensure analytical consistency, cell cultures were maintained under tightly controlled conditions, and both proteomic and spectroscopic datasets underwent rigorous quality control, including spectral assessment and reproducibility checks.

## Material and methods

### Cell culture and treatment

Two HL cell lines, L-428 and L-540, were used in this study: L-540 carries the wildtype p53 gene, whereas L-428 harbors a p53 mutation. Both cell lines were maintained in suspension culture. The structure of replicates is given in Table 1. In general, cell cultivation was initiated from in-house cryopreserved (cryo) stocks and originally obtained from the DSMZ. To assess the impact of the cultivation starting point on L-540 cells for proteomics analysis, they were also cultured from a living cell suspension, delivered at room temperature by the DSMZ. Accordingly, SERS measurements were performed on two cell lines: L-540 (cryo-recovered) and L-428. In the proteomics workflow, L-428 was compared with L-540 profiled in two distinct conditions, cryo-recovered (cryo) and actively cultured (living). For each biological replicate, two or three technical replicates were prepared, following a bottom-up proteomic workflow, and each technical replicate was measured in triplicate. A complete overview of manufacturers, devices, and substances is given in Electronic Supplementary Material Tab. S7–S9.

**Table 1:** Overview of the used biological, technical, and measurement replicates for the different cell types. *Cryo*, cryo-conserved L-540; *Living*, living cell culture L-540

Hodgkin lymphoma cell line	Analytical technique	Biological replicate	Technical replicate	Measurement replicate	Total number of individual measurements
L540 cryo	Proteomics	2	2/2	3	6/6
L540 living		2	2/3	3	6/9
L428 cryo		3	3/2/3	3	9/6/9
L540 cryo	SERS	3	2	≥ 50 cells	≥ 300 cells
L428 cryo		3	2	≥ 50 cells	≥ 300 cells

Cells were handled under sterile conditions in a laminar-flow biosafety cabinet and cultivated in RPMI-1640 supplemented with 20% fetal calf serum (FCS), 1 mM sodium pyruvate, and 1% (v/v) penicillin/streptomycin. Cells were maintained at 37 °C in a humidified atmosphere containing 5% CO_2_ and routinely monitored for viability and contamination. Media were replaced every 2–3 days, and cells were not passaged for more than 10 passages before treatment. For both SERS and proteomics experiments, treatment was initiated by adding 5 µM resveratrol [47, 48] to groups R (resveratrol-treated) and RE (resveratrol + etoposide treated) at the beginning of the incubation period. After 72 h of incubation, 25 µg/mL etoposide [49] was added to groups E (etoposide-treated) and RE. The C group served as untreated control. All four groups (R, RE, E, and C) were harvested 24 h after etoposide addition, resulting in a total incubation time of 96 h for all groups.

### SERS measurement

The SERS method was established using CaF_2_ slides and self-synthesized ascorbic acid/sucrose reduced 40 nm gold nanoparticles (Au-NPs) [50]. These nanoparticles were prepared according to standardized protocol and characterized previously by SEM and UV-Vis (Electronic Supplementary Material Fig. S1). The harvested cell suspension was washed with phosphate-buffered saline (PBS), fixed in 4% (v/v) formaldehyde for 20 min at room temperature, washed again in PBS, and in the final washing step rinsed with reverse osmosis (RO) water and centrifuged for 5 min at 400 × g. The fixed cells were resuspended in aqueous Au-NP colloid (1:1) and finally spotted and dried on a CaF_2_ slide. Per spot, a minimum of 50 spectra were measured. For each of the three biological replicates, this technical sample preparation was performed twice, yielding a minimum of 300 spectra per treatment condition. The measurement was performed on a Bruker Senterra Raman microscope (50× objective, NA 0.75, 785 nm laser, 4 × 5 s @ 25 mW). 

### Sample preparation for bottom-up proteomics

Cell samples described in section “Cell culture and treatment” were processed by applying a bottom-up proteomics approach. Cells were collected by centrifugation at 500 × g for 5 min, washed twice with 50 mM triethylammonium bicarbonate (TEAB) buffer, and after a final centrifugation at 5000 × g for 5 min, the cell pellets were stored at −80 °C. For protein extraction, pellets were lysed in 8 M urea in 50 mM TEAB containing protease inhibitor cocktail (5 µL per sample). Lysates were kept on ice, vortexed periodically for 2 h, and finally centrifuged at 14,000 × g for 15 min at 4 °C. Supernatants containing soluble proteins were transferred to low-binding tubes and stored at −80 °C. Protein concentrations were determined using the bicinchoninic acid (BCA) assay with bovine serum albumin standards (0–2000 µg/mL). Absorbance was measured at 562 nm after 30 min incubation at 37 °C.

For the bottom-up approach, cell lysates were processed using Microcon™ filter units washed three times with 8 M urea in 50 mM TEAB. Aliquots containing 20 µg total protein were loaded, adjusted to 300 µL with 8 M urea, and centrifuged at 10,000 × g for 20 min. Proteins were reduced with 20 mM dithiothreitol (50 µL, 37 °C, 30 min) and alkylated with 60 mM iodoacetamide (50 µL, 25 °C, 30 min, dark). Filters were washed twice with 50 mM TEAB. Digestion was performed with LysC/trypsin (1:25 enzyme-to-protein ratio) in 1 M TEAB for 16–18 h at 37 °C and 300 rpm. Peptides were recovered by centrifugation and washed three times with 50 mM TEAB. Protease activity was quenched by adding trifluoroacetic acid (TFA) to 1% (v/v). Samples were dried under vacuum at 45 °C and stored at −80 °C.

Dried peptides were resuspended in 1% TFA, vortexed, and sonicated before desalting with ZipTips™. Tips were conditioned with 80% acetonitrile (ACN) and washed with Milli-Q water. Peptides were bound by aspirating the sample ten times, washed, and eluted with 90% ACN. Eluates were concentrated under vacuum at 30 °C and stored at −80 °C. For nano-LC-MS/MS, peptides were reconstituted in 1% formic acid and sonicated, and 10 µL was transferred to nano-HPLC vials. Detailed method parameters can be found in the Electronic Supplementary Material Tab. S1.

### Data analysis

#### SERS

For the analysis of SERS spectra, we applied a Python-based workflow implementing twelve linear and non-linear machine learning classifiers combined with nested cross-validation. The evaluated models included LDA with various dimensionality reduction techniques, SVM, and logistic regression using two types of regularization (L1 and L2), as well as tree-based algorithms such as decision trees, random forests, and gradient-boosted decision trees (GBDT) [29, 45]. Prior to model training, spectral preprocessing was performed, including baseline correction, smoothing, and normalization, as described by Lilek et al. [45]. A quality control procedure was applied to remove spectra of insufficient quality.

Model training and evaluation were performed using nested cross-validation to ensure a strict separation between hyperparameter optimization and performance estimation. The hyperparameter search spaces were defined conservatively, based on prior methodological studies, to avoid overly flexible model configurations. This validation strategy minimizes information leakage and provides an unbiased estimate of generalization performance [45]. Model performance was primarily assessed using generalization accuracy and receiver operating characteristic (ROC) analysis. Additional evaluation parameters like the confusion matrix or F1 score and detailed settings are provided in the Electronic Supplementary Material Tab. S3 and Tab. S4. The biological interpretation of the coefficients of the machine learning model was carried out based on the assignments reported in the literature. The code for data analysis can be found on GitHub.^1^

#### Proteomics

We used MaxQuant (v2.0.3.0) on the Galaxy platform [51] for protein identification and label-free quantification. As the protein search database, FASTA files were downloaded from UniProt (14.07.2025) and trypsin was used for in silico digestion. Carbamidomethyl (C) was set as fixed, and N-term acetylation was used as variable modification. For identification, the false discovery rate (FDR) was set to 0.01, with MaxQuant using the target decoy approach for this [41]. The second peptide and the Match between Runs [41] option were disabled to reduce the number of protein ID transfers across the entire sample analyzed. Detailed configuration parameters can be found in the Electronic Supplementary Material Tab. S2. As a post-processing procedure, which included filtering, statistical testing, and visualization, we used R (v4.2.2) and RStudio (v2022.12.0) and an established automated workflow [35]. In the first step, proteins labelled as contaminants, reverse hits, or identified only by a modification site were removed, as recommended by Tyanova et al. [41]. Then LFQ values were log_2_ transformed. For quality control, we used PTXQC [33] and visualization techniques suggested by Schessner et al. [52]. Reproducibility was assessed based on the number of quantified proteins across three biological replicates for L-428 and two biological replicates each for the cryopreserved and living L-540 cultures (ggplot2 v3.5.2). To improve the performance on differential expression analysis (DEA), we imputed the data using llsImpute from the pcaMethods package (cluster size 10; all variables) as suggested by Välikangas et al. [42]. Data were not normalized because all datasets were processed in one single MaxQuant run [53]. For DEA, we used DEqMS [54] as suggested by Peng et al. [53]. The code for data analysis can be found on GitHub.^2^

For biological interpretation, a semi-targeted data analysis strategy was applied. Protein identification and quantification were performed in an untargeted, data-dependent acquisition (shotgun) mode, whereas subsequent statistical evaluation was restricted to a predefined panel of around 70 biomarker candidates compiled from the literature and found to be significantly altered across the four treatment conditions. This approach therefore combines unbiased proteome acquisition with focused, hypothesis-driven interpretation. The complete list of selected proteins, together with supporting literature, is provided in the Electronic Supplementary Material Tab. S6.

The proteomic and SERS data were processed and evaluated independently. Their relationship was then assessed at the level of subsequent biological interpretation.

## Results and discussion

### SERS-based molecular profiling and classification

**Fig. 1:**
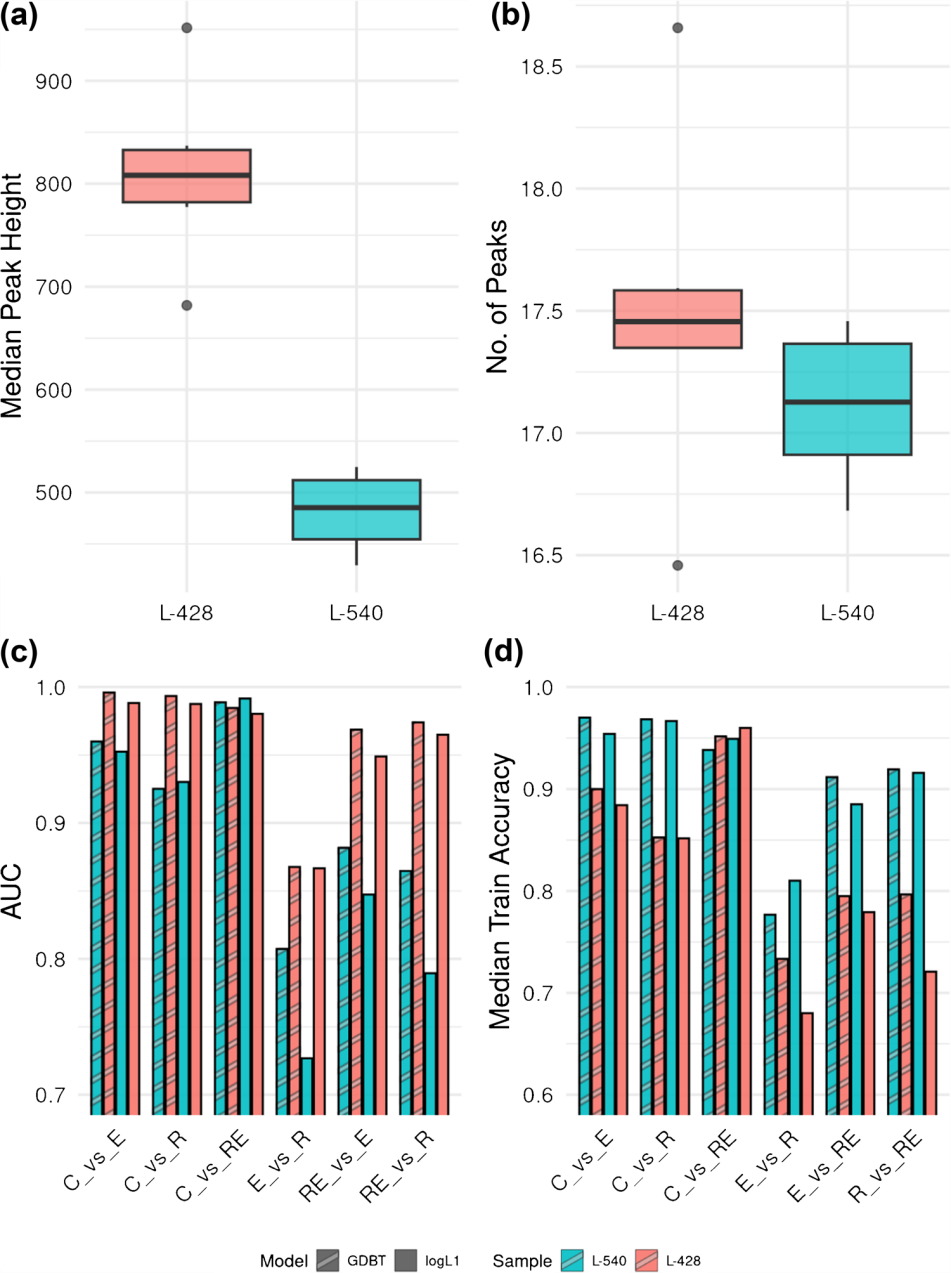
Data analysis of the Raman spectroscopic measurements of L-540 and L-428 cells. **a** Boxplots for the median peak height of the spectra used [a.u.]. **b** Number of detected peaks for the spectra used. **c**, **d** Median AUC and training accuracy for logistic regression with l1 regularization (logL1) and gradient-boosted trees (GBDT) for binary classification of different treatments (C, control; E, etoposide; R, resveratrol; RE, combined therapy with etoposide and resveratrol)

The initial step in the classification and differentiation of Raman spectroscopic data was to ensure the overall quality of the spectra [32]. As demonstrated in Fig. 1a, b, the number of peaks for both L-540 and L-428 is in the same range, averaging around 17–18 peaks. The median peak height exceeds the internally defined threshold. A notable distinction in peak height is evident between L-428 and L-540; nevertheless, within each cell line, the peak intensities exhibit a high degree of consistency. The distribution of peak heights (see Electronic Supplementary Material) exhibits a right-skewed pattern for all datasets, as previously reported by Lilek et al. [45], suggesting that spectra with lower median intensities are over-represented. Such skewness can influence the sensitivity of subsequent analyses and therefore requires appropriate statistical treatment. The results of the quality control (QC) analysis demonstrate that the Raman spectra across all datasets meet the requisite quality criteria in terms of both intensity and the number of peaks. Consequently, these spectra are suitable for subsequent data analysis.

To evaluate the effectiveness of the binary ML classifiers on spectral data, we compared all ML models based on generalization accuracy and ROC curves using nested cross-validation (Fig. 1c, d). LDA combined with different dimensionality reduction techniques such as PCA never reached the best classification performance achieved by other methods, such as logistic regression with L1 regularization (logL1) regardless of the cell line, treatment, or dimensionality reduction approach used (e.g., for L-540 RE-E/RE-C AUC PCA-LDA 0.98/0.81; AUC logL1 0.99/0.85). As demonstrated in previous studies [29, 45], L1 regularization outperformed L2 regularization for logistic regression and SVM (support vector machine) in most cases, which was also observed in the present study (see Electronic Supplementary Material for detailed results).

For the next comparison step, we focused on the two methods that performed best in earlier studies [29, 45]—logistic regression with L1 regularization (logL1) and gradient-boosted decision trees (GBDT). Since they achieved the highest accuracy among the tested models, we used them for a more detailed assessment of classification performance (Fig. 1c, d). The highest accuracy and AUC values were reached when comparing untreated control to treated cells. For L-428, the C–E comparison yielded an area under the curve (AUC) of 0.996 and an accuracy of 0.970 (GBDT), while C–R reached an AUC of 0.993 and an accuracy of 0.968. These results indicate clear spectral alterations following the treatment. Similarly, C-RE and GBDT attained an AUC of 0.9865 for the L-428 cell line. For L-540, most discriminative comparisons were C–RE (AUC 0.99; accuracy 0.96, logL1) and C–E (AUC 0.88; accuracy 0.95, logL1). The lowest values for accuracy and AUC were obtained for binary comparison of treated cells, especially for the E-R comparison (L-540 logL1: accuracy 0.68, AUC 0.73; L-428 GBDT: accuracy 0.78, AUC 0.87), suggesting minor spectral differences between the R, E, or RE treated cells.

As the classification task became more challenging, and the differences in cell status due to treatment dropped, greater inequalities in performance between logL1 and GBDT were evident among the models. For example, L-540 RE-R comparison (AUC logL1: 0.79; GBDT: 0.86; accuracy logL1: 0.72, GBDT: 0.80) and L-540 E-R comparison (AUC logL1: 0.73; GBDT: 0.81; accuracy logL1: 0.68; GBDT: 0.73). This suggests that non-linear, tree-based models are better suited for this task. This finding is consistent with the results reported by Lilek et al. [45], who indicated that advanced machine learning approaches are particularly advantageous for more complex and/or extensive datasets.

Despite the slightly lower performance of logL1 in complex cases, its inherent sparsity [55] leads to models that include only the most relevant features, thereby improving interpretability [45, 55] that is crucial for biological interpretation. In comparison, the GBDT models were interpreted using SHAP values [56], while their global feature importance reflects only mean absolute contributions and may therefore obscure heterogeneous sample-specific effects [55, 56].

Taken together, strong classification performance was achieved through binary comparison of control and treated cells. Nevertheless, the median AUC and accuracy for L-540 (AUC 0.889; accuracy 0.815) were slightly lower than for L-428 (AUC 0.973; accuracy 0.932), which may reflect smaller treatment-induced spectral differences, increased biological heterogeneity, or a reduced SERS activity (Fig. 1c, d). These results confirm that our standardized SERS-ML workflow, that carefully considered key methodological aspects such as regularization strategy, feature selection, and validation design [45], is well suited for classification tasks in SERS.

A biological interpretation was performed based on logL1 coefficients, using literature to assign bands to the most relevant features. For better readability, we have omitted references to literature in this section. Detailed results can be found in the Electronic Supplementary Material Tab. S5.

**Fig. 2:**
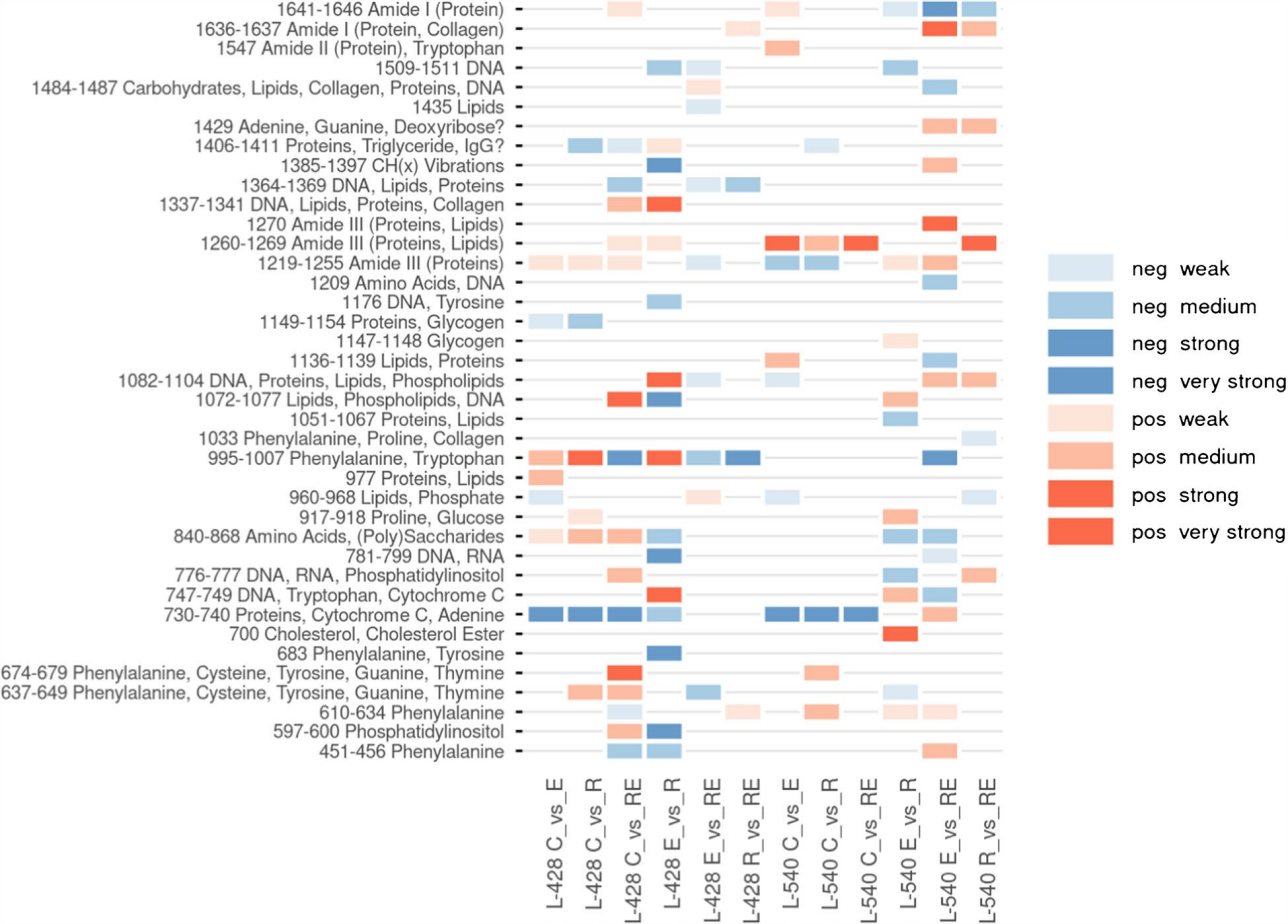
Heatmap of the most important logL1 coefficients for L-540 and L-428 cells. Blue shades indicate negative coefficients, while red shades indicate positive coefficients. The intensity of the color corresponds to the absolute effect strength, which is classified into four categories based on the absolute values of the logL1 coefficients (|*y*|): weak (|*y*|< 0.10), medium (0.10 ≤|*y*|< 0.30), strong (0.30 ≤|*y*|< 0.80), and very strong (|*y*|≥ 0.80). The columns represent the binary comparison for control and treated cells (C, control; E, etoposide; R, resveratrol; RE, combined therapy with etoposide and resveratrol). A detailed table and literature for the band assignment can be found in the Electronic Supplementary Material Tab. S5

For the L-428 cell line (Fig. 2), the most prominent spectral features were observed in treated cells around 1000 cm⁻^1^, corresponding to phenylalanine and tryptophan vibrations. Additional characteristic features occurred near 1110 cm⁻^1^, attributed to DNA and phospholipids, and at approximately 790 cm⁻^1^, associated with DNA backbones. When comparing control to treated samples, distinct differences became evident. In the C–RE comparison, control cells showed higher intensities in features assigned to phenylalanine and tryptophan (995–1007 cm⁻^1^), cytochrome c and adenine (730–740 cm⁻^1^), and protein-related amide III and aromatic amino acid vibrations (1219–1255 cm⁻^1^). Additional control-weighted features appeared near 451–456 cm⁻^1^ (phenylalanine) and 1189 cm⁻^1^ (DNA). In contrast, the RE-treated group displayed stronger signals around 674–679 cm⁻^1^ (amino acids and DNA), 1072–1077 cm⁻^1^ (lipids, phospholipids, and DNA), 776–777 cm⁻^1^ (DNA/RNA and phosphatidylinositol), and 840–868 cm⁻^1^ (proline and polysaccharides). In the C–E comparison, control cells exhibited upregulated features assigned to proteins, cytochrome c, adenine (730–740 cm⁻^1^), lipids or phosphate groups (960–968 cm⁻^1^), and glycogen- or protein-related features at 1149–1154 cm⁻^1^. In contrast, etoposide-treated cells showed prominent features near 977 cm⁻^1^ (proteins and lipids), 995–1007 cm⁻^1^ (phenylalanine and tryptophan), and 1219–1255 cm⁻^1^ (amide III proteins). The C–R comparison revealed pronounced differences as well. Protein- and cytochrome c–related features around 730–740 cm⁻^1^, as well as glycogen- and lipid-associated signals at 1149–1154 cm⁻^1^ and 1406–1411 cm⁻^1^, were more intense in control cells. Resveratrol-treated samples, however, showed enhanced signals for phenylalanine, cysteine, tyrosine, guanine, and thymine (637–649 cm⁻^1^) and for phenylalanine and tryptophan (995–1007 cm⁻^1^). When comparing the combined and etoposide-treated groups (RE–E), major differences were observed at 995–1007 cm⁻^1^ (phenylalanine and tryptophan), which were strongly upregulated under etoposide treatment. Weaker signals in the 637–649 cm⁻^1^ (aromatic amino acids), 1435 cm⁻^1^ (lipids), and 1364–1369 cm⁻^1^ (DNA, lipids, and proteins) regions were also observed in the E-treated condition. For the RE treatment, signals at 1006 cm⁻^1^ appeared more intense. The E–R comparison, which yielded the lowest classification performance across models (Fig. 1c, d), still revealed several differential features. Etoposide-treated cells displayed higher intensities in DNA/RNA-related vibrations (781–799 cm⁻^1^), in phospholipid and DNA regions (1072–1077 cm⁻^1^), and in aromatic amino acids near 683 cm⁻^1^ and 995–1007 cm⁻^1^ (phenylalanine). In contrast, resveratrol treatment was associated with stronger contributions from DNA, proteins, and lipid features (1082–1104 cm⁻^1^), unassigned feature near 461 cm⁻^1^ and 1540 cm⁻^1^, collagen-/protein-related vibrations (1337–1341 cm⁻^1^), and DNA/tryptophan/cytochrome c signals at 747–749 cm⁻^1^.

In the L-540 cell line (Fig. 2), the overall spectral intensities were lower than in L-428, but several characteristic trends were consistent. In the treated cells, strong contributions were seen for amide I and amide III vibrations (1260–1270 cm⁻^1^ and 1641–1646 cm⁻^1^), cytochrome c and adenine (730–740 cm⁻^1^), and phospholipid-associated features around 1270 cm⁻^1^. In the C–RE comparison, amide III and lipid features (1260–1269 cm⁻^1^) were more intense in treated cells, whereas cytochrome c and adenine (730–740 cm⁻^1^) were predominant in controls. For the C–E comparison, control cells showed more intensive features corresponding to cytochrome c and adenine (730–740 cm⁻^1^), amide III and aromatic amino acids (1219–1255 cm⁻^1^), an unassigned feature near 908 cm⁻^1^, and proline (1039 cm⁻^1^). In etoposide-treated samples, the amide III and lipid region (1260–1269 cm⁻^1^), amide II and tryptophan (1547 cm⁻^1^), and lipid–protein features (1136–1139 cm⁻^1^) were more prominent. In the C–R comparison, control features were dominated by the 730 cm⁻^1^ band (proteins, cytochrome c, adenine), while resveratrol-treated cells showed stronger contributions from 1260–1269 cm⁻^1^ (amide III proteins, lipids), 610–634 cm⁻^1^ (phenylalanine), and 674–679 cm⁻^1^ (aromatic amino acids including cysteine, tyrosine, guanine, thymine). The RE–E comparison revealed an overrepresentation of amide III proteins and phospholipids (1270 cm⁻^1^) in RE-treated cells, with unassigned features at 1497 cm⁻^1^ of unknown origin. Additional differences were noted in the 1636–1637 cm⁻^1^ region (proteins and lipids/collagen), more intense in RE-treated samples, while in etoposide-treated cells, features at 1641–1646 cm⁻^1^ (amide I), 995–1007 cm⁻^1^ (phenylalanine and tryptophan), 1385–1397 cm⁻^1^ (CH/CH₂ vibrations), and 1484–1487 cm⁻^1^ (carbohydrates, lipids, collagen, proteins, DNA) were more pronounced. In the RE–R comparison, the RE-treated group exhibited enhanced signals in amide III proteins and lipids (1260–1269 cm⁻^1^), adenine/guanine/deoxyribose (1429 cm⁻^1^), and DNA–lipid–protein regions (1082–1104 cm⁻^1^). Conversely, the resveratrol-treated cells showed stronger contributions from amide I proteins (1641–1646 cm⁻^1^), unassigned features at 563 cm⁻^1^, and phosphate/lipid features (960–968 cm⁻^1^). Finally, in the E–R comparison, etoposide-treated cells were characterized by higher intensities near 1509–1511 cm⁻^1^ (DNA), 560 cm⁻^1^ (unassigned), and 995–1007 cm⁻^1^ (phenylalanine and tryptophan). Resveratrol-treated samples displayed stronger features at 700 cm⁻^1^ (cholesterol and cholesterol ester), 1497 cm⁻^1^ (unassigned), 1641–1646 cm⁻^1^ (amide I proteins), and 917–918 cm⁻^1^ (proline and glucose).

Based on these observations, consistent patterns across the cell lines were further assessed through the comparison of the most relevant logistic regression coefficients. Positive coefficients, i.e., features weighted toward the control samples, were mainly observed in the adenine/cytochrome c spectral region, indicating higher contributions of nucleic acid and mitochondrial-associated signals [7, 8, 45]. This decrease after treatment is consistent with reduced ATP content and impaired mitochondrial activity. Signals attributed to amide III and amide I vibrations were generally enhanced in the treated groups and more pronounced in p53 WT L-540, suggesting protein conformational changes and partial unfolding [6, 7, 45]. A prominent and treatment-sensitive feature appeared around 995–1007 cm⁻^1^, assigned mainly to phenylalanine. Phenylalanine was consistently strong in all datasets and showed treatment-dependent shifts: in both L-428 and L-540, but particularly in L-428, this band exhibited positive coefficients in treated cells, indicating an increased contribution of phenylalanine-containing proteins. The pronounced intensity under etoposide and combined RE treatment in L-428 supports further its role as a key marker of protein and membrane alterations. The observed changes likely reflect conformational stress or membrane reorganization, consistent with previous Raman studies linking aromatic amino acid features to apoptosis and oxidative damage [30, 45].

### Proteomics

#### Reproducibility of the proteomics experiment

Prior to the reproducibility and repeatability analysis, which was based on RSD values and protein intersections, the quality of the data was assessed using PTXQC in combination with the established visualization strategies for bottom-up proteomics [52]. This assessment included, among others, evaluation of global intensity distributions, protein intersections, relative standard deviations (RSD), inter-replicate correlations, and PCA. No systematic outliers or batch-related deviations were detected across samples. Comparable global intensity distributions and consistent sample-level profiles across all conditions indicated stable LC-MS/MS performance. Biological reproducibility was supported by high inter-replicate correlation coefficients (*r* = 0.95–0.99 in L-540 cells) and median coefficient of variation (CV) values predominantly below 20%. PCA demonstrated the tightest clustering for repeated LC-MS/MS measurements of the same sample within each cell line, indicating high instrumental reproducibility. Technical replicates consistently clustered with slightly greater dispersion, reflecting minimal variability in sample preparation. As expected for independent biological samples, biological replicates are also grouped according to treatment condition, although with comparatively higher dispersion. Detailed reproducibility metrics, including PCA plots, correlation matrices, and CV distributions, are provided in the Supplementary Material.

**Fig. 3:**
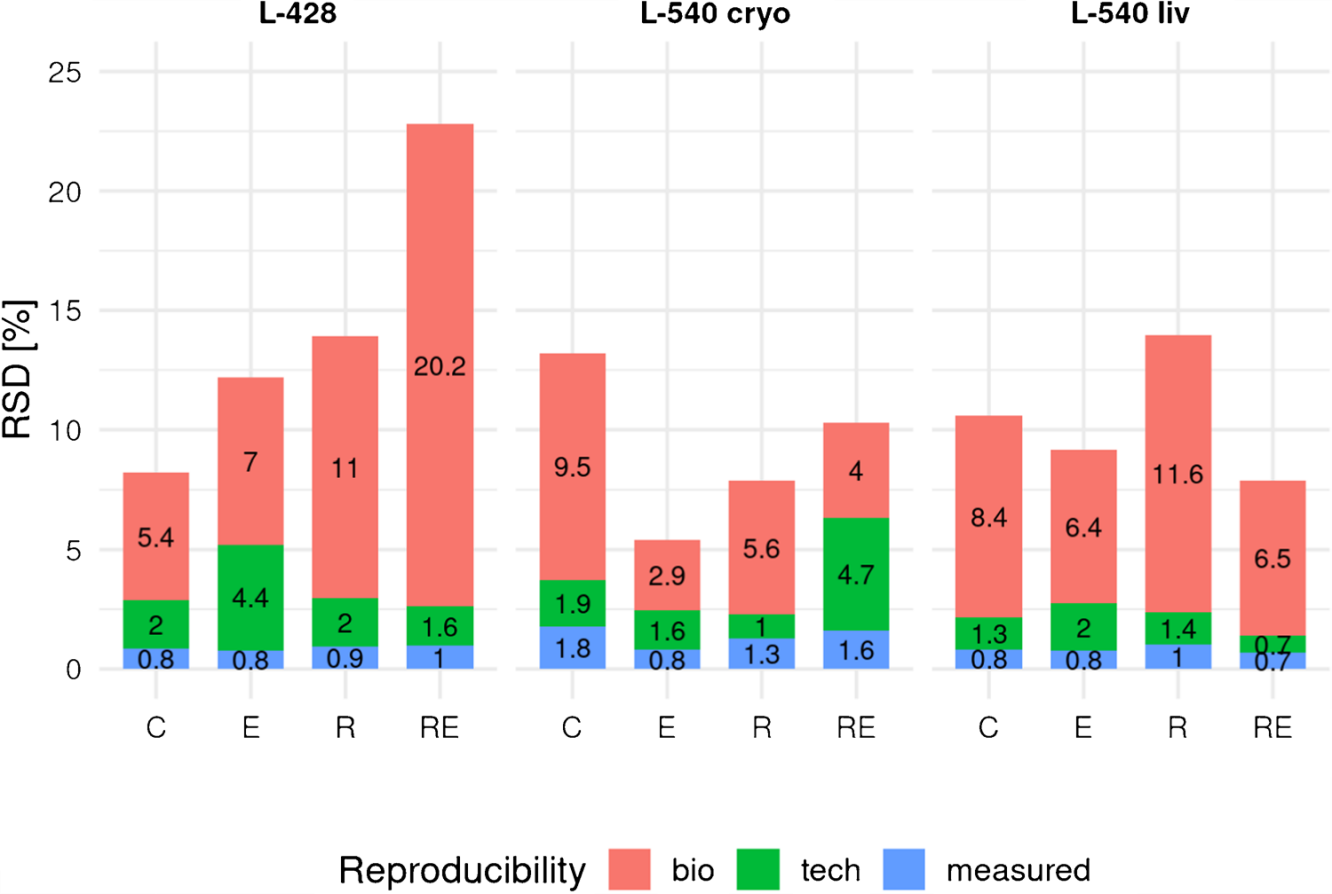
Reproducibility of the number of protein identifications for biological (bio), technical (tech), and measurement (measured) replicates for L-428 and L-540, living and cryo. The relative standard deviation (RSD) for control (C), etoposide (E), resveratrol (R), and RE (combined treatment with resveratrol and etoposide) treatment, based on the number of quantified proteins, is shown

Consistently with these QC results, the reproducibility analysis revealed low technical and measurement variation across all conditions and cell lines, confirming the robustness of the sample preparation and the nano-LC-MS/MS workflow (Fig. 3). The low RSD values (< 2%) for measurement replicates indicate a high stability of the instrumental performance and data processing pipeline, which was also shown in previous studies [35]. Slightly higher variability observed for sample preparation (up to 4.7%) reflects the expected experimental variation introduced during protein extraction, digestion, and sample handling (technical reproducibility). On the biological level, higher RSD values (median 6.7%) indicate higher intrinsic biological heterogeneity between treatment conditions and cell culture replicates. This is in line with previous studies [35] reporting that biological variability typically exceeds the technical and measurement variance in quantitative proteomics experiments. Notably, for the L-428 RE group, a markedly higher RSD of 20.2% was observed at the biological level. This increase can be attributed to one replicate, exhibiting a lower number of quantified proteins compared to the other two. As this biological replicate of L-428 cells (across all four treatments) was prepared at a different cultivation timepoint than the other two replicates, it more realistically represents biological variability.

**Fig. 4:**
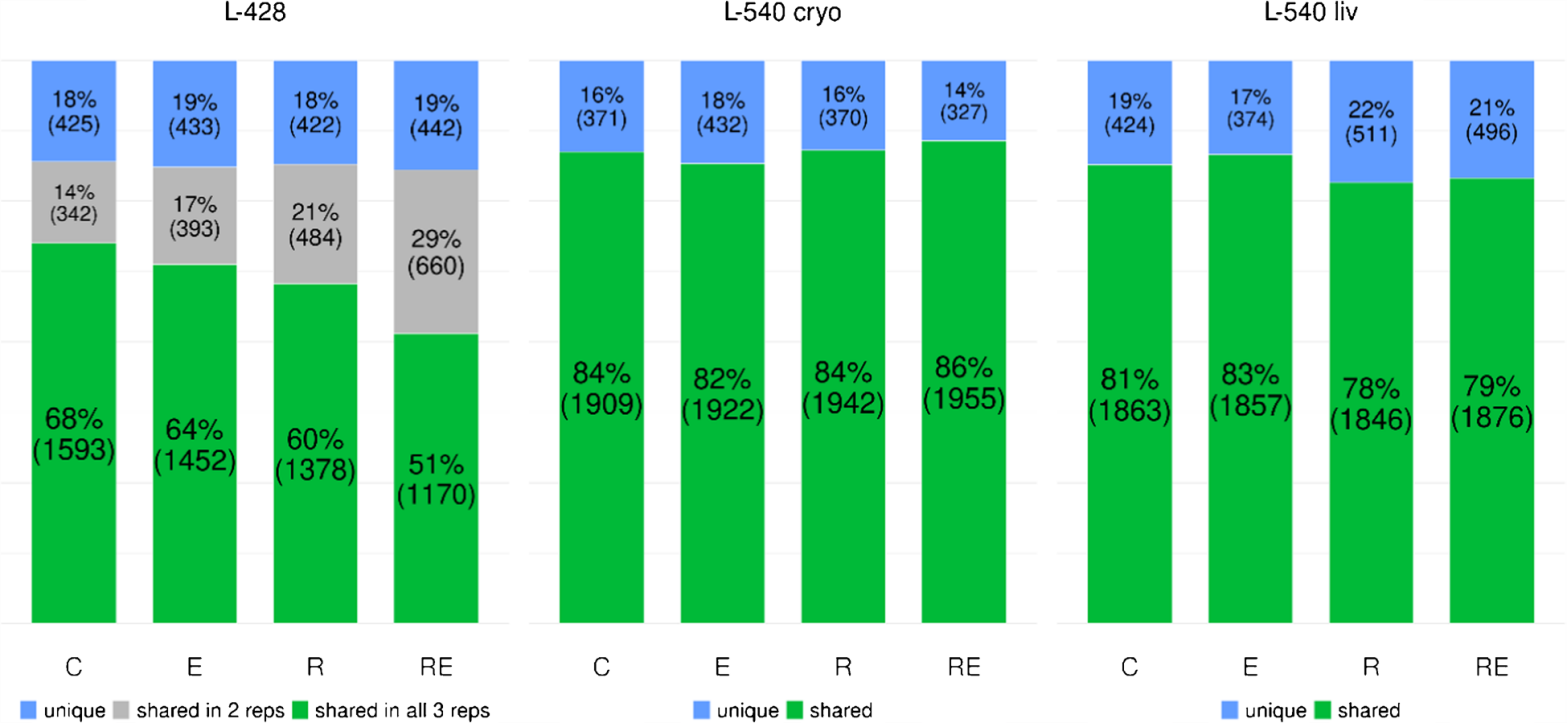
Reproducibility of protein quantifications in L-428 and L-540 cells under cryopreserved (cryo) and living (liv) conditions. Stacked barplots show the absolute counts and relative proportions of proteins detected across biological replicates. For L-540 (liv and cryo), reproducibility was summarized as 'unique' (proteins quantified in only one replicate) and shared (proteins quantified in both replicates). For L-428, the three replicates were categorized as unique (quantified in only one replicate), shared by two (quantified in exactly two replicates), or shared by all three (quantified in all replicates). The experimental conditions included control (C), etoposide (E), resveratrol (R), and a combined treatment of resveratrol and etoposide (RE)

Direct comparison of the quantified proteins based on UniProt accession numbers (Fig. 4) between treatments followed the same trend as the RSD analysis. The overlap of identified proteins was highest in L-540 cryo culture (82–86%), followed by L-540 living culture (78–83%) and L-428 (51–68%), indicating that sample preservation and handling affect the overall proteome coverage. For L-540, the overlap remained consistent between control and treated cells. This is not the case for L-428, where smaller overlaps, especially for the RE-treated cells, suggest treatment-specific alterations in protein abundance rather than methodological inconsistency. The number of quantified proteins reflected the same pattern, with the highest mean values in L-540 cryo samples (2119 proteins), slightly lower in L-540 living culture (2086 proteins), and the lowest in L-428 (1872 proteins). A direct comparison of L-540 living and L-540 cryo samples revealed an overlap of 58–62% (not shown here) in the quantified proteins. This smaller intersection is similar to the overlap observed in L-428, demonstrating that sample preservation and handling can significantly impact proteome coverage, even in datasets that otherwise exhibit high consistency within conditions.

In summary, both the relative standard deviation and protein overlap analyses, supported by prior quality control and global data inspection, demonstrate high analytical reproducibility and repeatability of the proteomics workflow and confirm that observed proteomic changes are primarily of biological rather than technical origin. The established protocol for bottom-up, nano-LC-MS/MS sample preparation was robust for all optimized steps—digestion, protein extraction, and desalting.

#### Selected proteomic findings and their corresponding SERS signatures

**Fig. 5:**
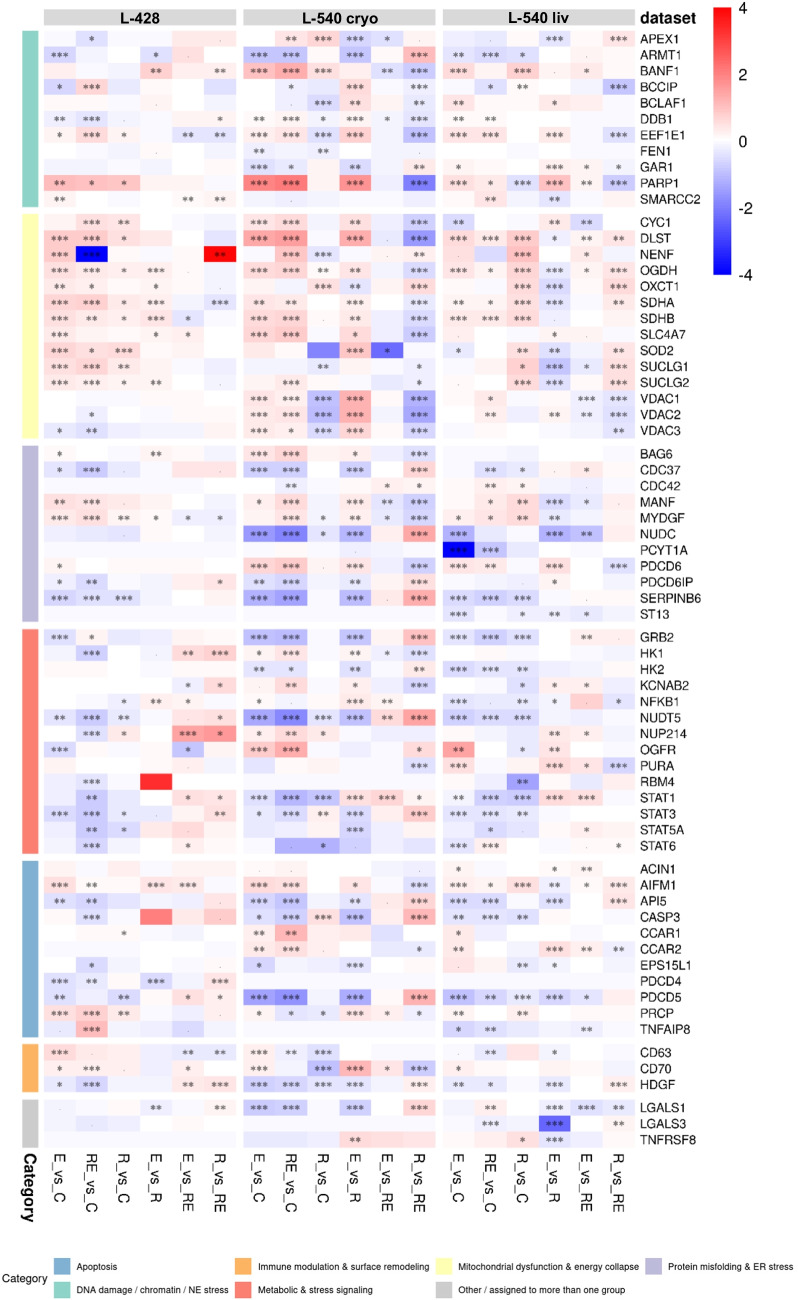
Heatmap of selected differentially expressed proteins in cell lines L-428 and L-540 (cryopreserved and living culture). Shown are log₂ fold changes for pairwise treatment comparisons (C, control; E, etoposide; R, resveratrol; RE, combined treatment with R and E). Color intensity indicates relative protein abundance changes (blue, downregulated; red, upregulated). Significance levels are annotated in each box: ***p < 0.001 | **p < 0.01 | *p < 0.05 |. *p* < 0.1 | blank, not significant. Column gaps separate datasets (L-428, L-540 cryo, L-540 liv). For each cell line, the proteins of interest are listed alphabetically in the corresponding major context category. For better visibility, log_2_ FC values higher than 4 were set to 4

We investigated treatment-induced changes in signaling, metabolism, and cell-fate regulation. A central outcome is the divergent stress-response behavior between the two cell lines, which correlates with their TP53 status. The comprehensive proteomic analysis across all independent biological replicates revealed both robustly conserved (consensus) and context-dependent (divergent) molecular responses to treatment with E, R, and their combination, RE. Applying a significance threshold of adj.*P* < 0.10 and |log_2_ FC|> 0.58 in the binary comparisons, a distinct pattern emerges that captures the dual nature of the RE response: a reproducible, multi-axis stress signature common to all systems, and additional, p53-dependent refinements that determine cellular fate. The results of the binary comparisons are visualized in a heatmap (Fig. 5). The cross-line and cross-treatment results are discussed in six major context sections: (1) DNA damage, chromatin remodeling, and nuclear envelope stress; (2) mitochondrial dysfunction and energy collapse; (3) protein misfolding, ER stress, and proteostasis; (4) metabolic and stress signaling regulation; (5) apoptosis and death signaling; and (6) immune modulation and surface remodeling. For better readability, we have omitted most of the references to literature in this section. Detailed results can be found in the Electronic Supplementary Material, Tab. S6.

##### DNA damage, chromatin remodeling, and nuclear envelope stress

Proteins associated with DNA damage signaling and chromatin stability were regulated in both Hodgkin lymphoma models after treatment with resveratrol and etoposide, with a more coordinated response in TP53-wildtype L-540 than in TP53-mutant L-428. In L-540 cryo, APEX1—the major AP-endonuclease of the BER pathway—was strongly induced by resveratrol alone (R vs C: log2FC + 0.64). Under etoposide, however, its expression was comparable to the untreated control, and the combined treatment (RE) abolished this R-driven induction, indicating that etoposide counteracts the resveratrol-driven upregulation of BER components. ARMT1, a PCNA-associated methyltransferase, implicated in post-damage chromatin remodeling, showed a clear downregulation in L-540 cryo under both E and RE (E vs C, −0.81; RE vs C, −0.88), consistent with reduced chromatin-adjustment capacity during accumulating DNA damage. Nuclear-structural remodeling followed similar trends: **BANF1**, which anchors chromatin to the nuclear lamina, was strongly upregulated under E and RE in L-540 cryo (E vs C, + 0.96; RE vs C, + 1.36), suggesting adaptive nuclear envelope restructuring upon checkpoints activated. **BCCIP**, a BRCA2-interacting factor required for homologous recombination, exhibited a more nuanced pattern in L-540, with elevation in etoposide-containing treatments only when compared to resveratrol (E vs R and RE vs R). This indicates overall stability of BCCIP levels, except for a resveratrol-specific suppression relieved in the presence of etoposide. A key hallmark of etoposide-induced DNA stress in L-540 was the pronounced activation of **PARP1**. As a primary mediator of the cellular response to topoisomerase II–mediated DNA breaks, its strong induction in L-540 cryo under E and RE (E vs C, + 1.96; RE vs C, + 2.11) aligns with the known activation of PARP1 upon etoposide exposure, reflecting extensive chromatin engagement and escalating damage signaling in the TP53-wildtype cells.

In L-428, the overall response was less coordinated. ARMT1 decreased only under E (–0.59), and APEX1 showed no significant regulation. **BCCIP** exhibited inconsistent modulation, suggesting secondary influences rather than a unified DNA damage response architecture. In contrast, PARP1 was consistently upregulated in L-428 across all treatments (E vs C, + 1.10; R vs C, + 0.91; RE vs C, + 0.98), indicating sustained chromatin-associated stress but lacking synchronized downstream remodeling consistent with impaired checkpoint regulation in the TP53-mutant background.

We additionally evaluated BCLAF1, EEFL1E, DDB1, FEN1, and GAR1, none of which showed differential expression under the treatment conditions. Taken together, L-540 exhibits a p53-linked shift from early BER activation (APEX1↑ under R) toward chromatin stress (ARMT1↓), nuclear envelope remodeling (BANF1↑), and robust PARP1 activation under etoposide. L-428, in contrast, shows inconsistent regulation with preserved PARP1 activation and reduced coordination of chromatin-associated pathways. These findings align with established mechanisms of etoposide-induced DNA damage and with reports describing modulatory effects of resveratrol on chromatin-associated stress responses.

These proteomic alterations are mirrored spectroscopically by attenuation of SERS bands associated with the porphyrin ring of cytochrome c, adenine, and DNA, together with increased amide and aromatic amino acid signals. The resulting shift toward protein- and amide-dominated spectra reflects the same transition captured by the proteome: from repair-oriented responses toward apoptotic commitment.

##### Mitochondrial dysfunction and energy collapse

The two HL models responded divergently to mitochondrial metabolism and demonstrated contrasting abilities to integrate stress. While TP53-wildtype L-540 exhibited a coherent response, TP53-mutant L-428 displayed, again, a more diverse pattern. A feature shared by both cell lines, however, was the upregulation of dihydrolipoamide–S–succinyltransferase (**DLST**), the E2 component of the α-ketoglutarate-dehydrogenase complex that promotes flux through the TCA cycle and thereby supports mitochondrial NADH production. In our study, DLST increased under both E and RE in L-428 (≈ + 0.86) and even more strongly in L-540 (+ 1.37 and + 1.57), suggesting enhanced TCA flux as an early compensatory response to genotoxic stress.

Other mitochondrial enzymes showed clearer genotype-specific behavior. In L-540, **OGDH** was consistently upregulated under E and RE, whereas L-428 showed no change. **SUCLG1** increased under RE in L-428, while in L-540 living it increased under resveratrol and decreased again upon addition of etoposide, indicating that E counteracts R-mediated enhancement of substrate-level phosphorylation. Several redox markers further distinguished the two lines. Primary mitochondrial superoxide dismutase (**SOD2**) converts superoxide into hydrogen peroxide and oxygen, thereby preventing ROS-driven damage to mitochondrial proteins and membranes. SOD2 showed a modest increase in L-428 (+ 0.67), whereas L-540 cryo displayed a more complex pattern (E vs R, + 0.95; RE vs C, −2.24). This may indicate that combined treatment compromises mitochondrial antioxidant defense in L-540 cells, potentially rendering cells more susceptible to ROS-dependent stress. **NENF**, a redox-linked growth factor, also showed pronounced line-specific regulation. In L-540 cryo, it increased moderately under resveratrol, consistent with a temporary adaptation to promote survival. In L-428, however, NENF rose under etoposide (+ 0.83) but decreased dramatically under RE (−4.0), suggesting that combined mitochondrial and genotoxic stress overrides NENF-mediated survival signaling. VDAC isoforms, which form the major metabolite and ion channels of the outer mitochondrial membrane and regulate ATP/ADP exchange and Ca^2^⁺ flux, were differentially regulated exclusively in L-540 cryo. VDAC1 and VDAC2 were lowest under resveratrol (− 0.96/− 0.97) but strongly increased whenever etoposide was present (+ 1.15 to + 1.30). VDAC3 showed a similar etoposide-associated increase without R-induced downregulation. This resveratrol-to-etoposide shift in VDAC1–3 indicates stress-dependent remodeling of mitochondrial membrane permeability.

A particularly clear, line-specific signal was observed in the complex II proteins **SDHA** and **SDHB**. SDHA was upregulated only in L-428 under E and RE, suggesting increased electron transport during genotoxic stress in p53-deficient cells. In contrast, SDHB was selectively upregulated in L-540 (E and RE in cryo; R in living), indicating a genotype-dependent modulation of complex II activity under metabolic and redox pressure.

Overall, mitochondrial protein signatures diverged between the two HL models. L-540 showed a stress-response characterized by strong TCA activation (DLST, OGDH), selective modulation of SUCLG1, loss of antioxidant defense (SOD2↓), NENF-linked pro-survival adjustment under R, and a pronounced etoposide-driven remodeling of VDAC1-3. In contrast, L-428 displayed only partial and less coordinated metabolic adaptations, including SDHA and SUCLG1, but lacked consistent redox or membrane remodeling. Together, these patterns indicate effective mitochondrial stress integration in L-540, whereas L-428 exhibits fragmented adaptations consistent with impaired p53-dependent checkpoint control.

Although CYT1, OXCT1, and SUCLG2 were not differentially expressed, the proteomic data of TCA cycle enzymes (DLST, OGDH, SDHA/B) and SOD2 align closely with the corresponding SERS profile. SERS revealed diminished cytochrome-c-associated bands, a hallmark of reduced mitochondrial redox activity. Moreover, newly appearing or intensified spectral features arising from guanine, thymine, lipids, phospholipids, and DNA indicate concurrent membrane and phospholipid remodeling, while enhanced amide I/III bands point to mitochondrial membrane permeabilization and protein structural rearrangements.

##### Protein misfolding, ER stress and proteostasis

Proteins involved in protein quality control, ER stress, and vesicular membrane remodeling revealed distinct patterns in the two models, with the pattern in TP53-wildtype L-540 cells showing broader pathway engagement than in TP53-mutant L-428 cells. Several chaperones and co-chaperone factors responded in a treatment- and line-specific manner. **BAG6**, a component implicated in cytosolic quality control and DNA damage signaling, was induced only in L-540 cryo under combined treatment (RE vs C). This observation is compatible with selective engagement of BAG6-linked proteostasis pathways in the TP53-wildtype cells under high stress, but the precise functional consequence (ERAD activation) remains to be confirmed. In contrast, the protease inhibitor **SERPINB6**, which is generally considered cytoprotective under proteotoxic and oxidative conditions, was reduced under etoposide in L-428 and consistently and strongly decreased in L-540 (average −1.32) whenever etoposide was present. This loss of inhibitory protection suggests that protease activity is less restricted under genotoxic stress, potentially facilitating irreversible proteome remodeling and cell death. The HSP90 co-chaperone, **CDC37**, which stabilizes and matures many signaling kinases, showed a similar stress-dependent attenuation. In L-428, it was downregulated under RE versus control, whereas in L-540 cryo, it decreased whenever etoposide was part of the treatment. Together with the cytoskeletal and ER stress linked factor **NUDC**, which was unchanged in L-428 but consistently downregulated in L-540 under E-containing conditions (down to about − 1.5), these findings suggest that p53-wildtype cells gradually break down chaperone and structural support networks once stress exceeds a repairable threshold. This contrasts with the usual view of upregulation of chaperones under moderate stress and is more consistent with a late phase of proteostasis during apoptosis. UPR- and ER-stress-associated mediators further supported this interpretation. **MANF**, an ER-localized stress-response factor, remained unchanged in L-428 but increased in L-540 under RE (RE vs C and RE vs E), indicating activation specifically under the combined treatment. The induction of **PDCD6** together with reduced **PDCD6IP** under RE in L-540 could reflect an altered ESCRT-adapter balance. This may favor vesiculation processes over repair in our system, a hypothesis consistent with ESCRT-involvement in late apoptosis but not uniquely diagnostic for it. Finally, the ER-membrane regulator **PCYT1A**, which controls phosphatidylcholine synthesis and thus membrane biogenesis, showed a striking suppression in L-540 living, being strongly downregulated under both etoposide and RE compared to the control (around −4.0). No such regulation was observed in L-428 or L-540 cryo. The strong PCYT1A suppression in L-540 living cells indicates a loss of ER-membrane renewal capacity under sustained genotoxic stress. This reinforces the concept that p53-wildtype cells shift from adaptive ER expansion to controlled membrane dismantling once apoptosis becomes unavoidable.

Taken together, L-540 integrates chaperone attenuation (SERPINB6↓, CDC37↓, NUDC↓), UPR activation (MANF↑ under RE), ESCRT remodeling (PDCD6↑, PDCD6IP↓), and loss of membrane biogenesis (PCYT1A↓ in living) into an ER-stress program that supports apoptotic progression. In L-428, by contrast, regulatory responses are more restricted and dominated by isolated decreases—such as SERPINB6 and CDC37—without the same multilayered convergence of distinct signal pathways.

These alterations are spectroscopically represented by increased amide I and III signals and enhanced aromatic amino acid vibrations, indicative of protein unfolding. Subtle increases in lipid- and phospholipid-associated SERS features, particularly in treated TP53-mutant cells, align with proteomic evidence for ESCRT-linked membrane remodeling and reduced membrane biogenesis.

##### Metabolic and stress signaling regulation

Metabolic–stress signaling in both Hodgkin lymphoma models was governed by two major regulatory axes: the GRB2–RTK–MAPK/PI3K node and the JAK/STAT network, forming a p53-dependent hub that links metabolic balance with apoptotic commitment.

In p53-competent L-540 cryo, **GRB2** was strongly suppressed under etoposide and RE, indicating collapse of RTK-derived MAPK/PI3K–AKT input during apoptosis initiation. **STAT** proteins showed parallel attenuation, with marked reductions under RE for STAT1 (–1.08) and STAT6 (–1.16), and similar decreases for STAT3 and STAT5A under RE or R. L-540 living displayed the same but weaker RE-dependent pattern. In contrast, p53-mutant L-428 showed unchanged GRB2 and only modest RE-specific STAT reductions (STAT1 − 0.75; STAT5A − 0.78), reflecting limited coupling between metabolic stress and transcriptional adaptation. Accordingly, RE enforces a coordinated mitogenic shutdown in L-540, but only a partial, unsustained response in L-428. In line with this trend, NF-κB in L-540 living was lower under RE compared to etoposide alone (E > RE), whereas L-428 showed no regulation, consistent with reduced signaling adaptability in the mutant background.

A second major signaling layer was formed by a three-tiered physicochemical stress-buffering module consisting of **NUDT5**, **SLC4A7**, and **KCNAB2**, integrating nuclear ATP regeneration (NUDT5), intracellular pH control (SLC4A7), and membrane ion stabilization (KCNAB2, the potassium channel pore-forming subunit). In L-540 (p53 WT), RE induced a compensation pattern characterized by SLC4A7 upregulation despite strong NUDT5 suppression (− 1.83), while KCNAB2 decreased (− 0.61), suggesting reliance on bicarbonate-driven pH buffering under declining nuclear ATP availability. In contrast, L-428 (p53-mutant) failed to induce SLC4A7 under NUDT5 loss, but increased KCNAB2 (+ 0.61), indicating a shift toward ion-channel–linked buffering. VDAC1–3, differentially regulated exclusively in L-540 cryo, aligned with this buffering module and supported stress-dependent remodeling of mitochondrial ion permeability.

Checkpoint signaling also diverged sharply between the two systems. **OGFR** showed a pronounced p53-dependent split. In L-540 (cryo and living), OGFR was induced by all treatments, with the strongest increases under R and RE (up to + 1.3 log₂FC, *p* < 10⁻^4^). In L-428, OGFR rose under etoposide but was strongly suppressed under RE (E vs RE − 0.85), reflecting ineffective checkpoint engagement in the absence of functional p53. In contrast, **PURA** was unchanged across all conditions (*p* = 0.00–0.01), indicating preserved nucleic acid regulatory scaffolding. Metabolic input into central carbon metabolism remained largely stable. Only **HK1** showed biologically relevant suppression in L-428 under RE (− 0.68), while HK2 remained unchanged, indicating limited metabolic adaptability in the mutant line. Nuclear transport responded in opposite directions: **NUP214** increased under stress in L-540 cryo, whereas L-428 showed a strong RE-driven loss (E vs RE + 1.44; R vs RE + 1.68), indicating NPC stabilization in p53-WT cells but collapsed in the mutant background. Finally, **RBM4** acted independently of the buffering triad and showed no RE-coordinated regulation and responded only to single agents: a strong but non-significant increase in L-428 under E vs R (+ 3.31) and a significant decrease in L-540 living under R vs C (−1.40) without RE-dependent coordination. Its behavior suggests non-integrated, treatment-specific RNA-processing modulation.

Overall, L-540 exhibits effective stress adaptation characterized by RE-driven suppression of mitogenic signaling and reinforcement of ATP buffering, pH control, nuclear transport, and growth restraint, whereas L-428 displays selective collapse of signaling, transport, and metabolic support under combined treatment.

SERS enrichment of phenylalanine and tryptophan vibrations in RE- and E-treated cells corroborates these proteomic indicators of metabolic stress, reflecting accumulation of aromatic intermediates and stress-associated alterations in membrane composition.

##### Apoptosis and death signaling

Analysis of apoptosis-related proteins revealed cell line–specific adaptations in checkpoint control and stress-response pathways, with the pattern showing broader pathway convergence in TP53-wildtype L-540 than in TP53-mutant L-428. The mitochondrial apoptosis-inducing factor **AIFM1**, which triggers large-scale DNA fragmentation during caspase-independent apoptosis, was elevated only in L-540 under etoposide, whereas it remained unchanged in L-428. This pattern is consistent with reports showing that severe genotoxic stress, such as topoisomerase II inhibition by etoposide, can trigger the release of AIF during mitochondrial permeabilization. The effector-caspase **CASP3** showed a reduced abundance in all etoposide-containing treatments in L-540 and, to a lesser extent, also in L-428 (only RE vs R). Because bottom-up proteomics does not distinguish between pro-caspase and the cleaved active enzyme, the reduced abundance is consistent with consumption or degradation during sustained caspase activation [36]. The decrease in L-540 thus supports a more advanced apoptotic state than in L-428, where CASP3 regulation was minimal. Several modulators of apoptotic sensitivity also showed genotype-specific behavior. The pro-apoptotic signaling amplifier **PDCD5**, which is known to promote p53-dependent caspase activation and nuclear condensation, was consistently and strongly reduced in both L-540 cultures whenever etoposide was present, suggesting that its contribution is limited to earlier phases of the response. **PRCP**, which has been implicated in cell survival and stress signaling through peptide processing and regulation of angiotensin/kinin pathways, was selectively induced in L-428 under RE but remained unchanged in L-540, which is consistent with a more compensatory rather than executive stress-response in TP53-mutant cells. **LGALS1** and **LGALS3**, two galectins which are frequently implicated in apoptosis modulation and chemoresistance in HL and other lymphomas, showed a distinctly genotype-dependent pattern. In L-540, LGALS1 was consistently downregulated whenever etoposide was involved, and LGALS3 was most strongly decreased under E vs R (−2.38). This is consistent with their known roles in supporting survival and dampening of apoptotic signals through receptor clustering, redox modulation, and interference with death-receptor pathways. However, their unchanged levels in L-428 suggest their retainment even under genotoxic pressure. Survival-associated mediators also separate the two models. **TNFAIP8**, an apoptotic factor frequently implicated in NF-ĸB-dependent apoptosis resistance that interferes with caspase activation and death-receptor signaling, decreased in L-540 under E but increased only in RE in L-428. Similarly, **TNFRSF8/CD30**, which can participate in apoptotic or survival signaling depending on cellular context [4], increased uniquely in L-540 E vs R and remained unchanged in L-428, indicating divergent surface-linked death signaling. In contrast, **API5**, a nuclear anti-apoptotic factor that inhibits E2F1-driven caspase-dependent cell death, was reduced in the RE condition in both models (RE vs C L-540: −0.88; L-428: 0.60). This indicates that the combined treatment with etoposide and resveratrol results in a loss of this protective mechanism.

Overall, L-540 integrates mitochondrial (AIFM1↑), caspase-related (CASP3↓), and modulatory (LGALS1/3↓, TNFAIP8↓) changes into an effective apoptotic execution program under etoposide, while both models share loss of the survival factor API5 under combined treatment. In contrast, L-428 exhibits isolated and occasionally opposing alterations (e.g., PRCP↑ and TNFAIP8↑) that do not converge on a unified execution pathway, reflecting impaired damage response integration in the TP53-mutant background.

SERS intensification of amide I/III and lipid–protein feature regions, together with loss of cytochrome-associated porphyrin and nucleotide signals, captures this apoptotic endpoint at the biochemical level, mirroring membrane and mitochondrial remodeling during cell death.

##### Immune modulation and surface remodeling

Beyond apoptosis, several proteins involved in immune signaling, glycan-mediated interactions, and membrane remodeling showed differences that reflect the distinct microenvironment and communication phenotypes of the two HL models. The immunomodulatory galectins, LGALS1 and LGALS3, whose role in apoptosis has already been discussed above, also contribute to immune evasion, T-cell suppression, and altered glycan interactions in HL. Both proteins were consistently reduced in L-540 under etoposide-containing conditions but remained unchanged in L-428. Because both galectins can regulate survival signaling and glycan-mediated cell interactions, their reduction in L-540 may contribute to the weakening of immune-protective cycles under genotoxic stress, whereas in the TP53-mutant L-428 the absence of modulation suggests that they maintain galectin-driven immune isolation. Additional surface and vesicle-associated markers further refined these patterns. **CD63**, a tetraspanin involved in the biogenesis of exosomes and vesicle transport, was upregulated only in L-428 under etoposide. In contrast, CD70, a ligand of the TNF-family that drives co-stimulation and immune activation, was selectively modulated in L-540, being downregulated under resveratrol and upregulated under both E vs R and RE vs R. This treatment-dependent regulation suggests that **CD70**-mediated immune communication is more sensitive in the TP53-wildtype cells. Finally, the secreted growth factor **HDGF**, which is often associated with immune modulation, angiogenesis and tumor-stroma interactions, was downregulated under etoposide and RE in L-540 but remained unchanged in L-428. Its suppression fits into the broader pattern of etoposide-driven remodeling of extracellular communication signals in L-540.

Taken together, immune- and surface-associated proteins distinguish the two HL models: L-540 modulates galectins and multiple communication receptors (LGALS1/3↓, CD70↑/↓, HDGF↓), whereas L-428 exhibits isolated changes such as CD63 without broader network adaptation.

These patterns indicate that immune and communication networks are more dynamically regulated in the p53-competent phenotype, whereas TP53-mutant cells have more static or dysregulated interactions with their microenvironment. A cross-reference with the apoptosis-associated behavior of LGALS1/3 and TNFRSF8 highlights that several of these mediators occupy functional interfaces between death signaling and immune modulation, underlining the diverse consequences of p53 status in HL stress responses.

This immune-surface remodeling is mirrored by SERS through enhanced lipid- and carbohydrate-associated bands, consistent with altered membrane composition and increased exposure of surface glycoconjugates typically associated with immune adaptation and extracellular communication changes.

The molecular stress signatures resolved in this section by proteomics and SERS are supported by independent biological validation obtained in a closely related experimental setting. Previously reported data demonstrated a pronounced TP53-dependent differential response of HL cell lines to etoposide treatment: TP53-wildtype L-540 cells responded faster and more robustly than TP53-mutant L-428 cells with respect to reduced proliferation and viability, induction of apoptosis and necrosis (absent in L-428), accelerated nuclear fragmentation, and highly significant differential expression of apoptosis-related genes (*p* < 0.001), including TP53, TNFα, HO1, and GADD45. Putative synergistic effects of resveratrol were observed for proliferation, nuclear fragmentation, and gene expression.

This study has inherent limitations. It was conducted exclusively in well-defined in vitro cell line models and did not include patient-derived samples. Furthermore, no formal computational multi-omics integration or feature-level data fusion was performed; instead, SERS and proteomics were evaluated independently and related through convergent biological interpretation. Future work will focus on the implementation of formal multi-omics integration. In addition, apoptosis-specific validation assays were not included in the current experimental design, and differences in biological replicate structure between the two analytical approaches limit direct cross-modal comparison at the individual replicate level. These constraints define important directions for future translational and integrative research.

## Conclusion

We developed and applied a methodological proof-of-concept workflow applying a LC-MS/MS-based bottom-up proteomics and surface-enhanced Raman scattering (SERS) into two classical Hodgkin lymphoma (HL) cell lines with distinct TP53 backgrounds. This approach enabled a systematic assessment of treatment-induced molecular stress responses, caused by single or combined treatment with etoposide and resveratrol, and addressed the translational need for technically standardized and biologically informative biomarker studies.

The biological impact of resveratrol’s chemosensitizing properties became particularly evident in combination therapy: RE consistently induced a stronger and more mechanistically coherent stress phenotype than either single agent, validating the biological relevance of our R, E, and RE comparisons and demonstrating the sensitivity and reproducibility of the established proteomic-spectroscopic workflow to subtle but synergistic treatment effects. Importantly, separating L-540 cryo and L-540 living cultures proved informative: multiple regulatory events were markedly weaker in living culture cells, whereas the cryo workflow delivered clearer and more reproducible molecular signatures. This distinction underlines that cryopreserved cultures provide a more stable and analytically robust representation of treatment-induced apoptotic processes.

The proteomics workflow yielded highly reproducible and quantitatively robust data across all treatment conditions, confirming the reliability of the standardized procedures for protein extraction and digestion. Further methodological refinements, such as the use of the latest mass spectrometric technologies with higher scan rates combined with optimized extraction workflows that enrich for proteins carrying specific post-translational modifications (PTMs), or enrichment-based data analysis, can substantially increase proteome completeness even further [57–59].

The high-quality SERS spectra, as confirmed by automated quality control, demonstrated the robustness of the measurements, although the overall lower intensity in L-540 posed additional challenges for classification and for the interpretation of logL1 coefficients. Among all tested models, the linear logL1 regression achieved the best balance of performance and biological transparency, whereas more complex tree-based models offered limited interpretability [45]. Manual band assignment remained time-consuming and highlighted the need for automated, database-assisted annotations to accelerate meaningful spectral interpretation. Based on the SERS observations, treated cells showed a decrease in nucleotide/cytochrome C features and an increase in amide and aromatic amino acid features. These spectral trends are consistent with DNA damage, mitochondrial dysfunction, membrane remodeling, and protein unfolding—molecular hallmarks of genotoxic or oxidative stress responses [60–63].

Taken together, the SERS and selected key-proteomic data converge on three interlocked pathways, DNA/chromatin stress, mitochondrial dysfunction, and proteostasis collapse, that collectively define the combined resveratrol/etoposide response. In TP53-wildtype L-540, these pathways align into a coordinated apoptotic program characterized by repair shutdown, metabolic repression, cytochrome-c release, and structural protein reorganization, all traceable by characteristic amide, aromatic, and cytochrome-associated Raman features. In TP53-mutant L-428, the same axes are only partially engaged, producing asynchronous or compensatory molecular signatures and retaining persistent nucleotide- and survival-linked spectral components. Thus, SERS provides a rapid chemically specific fingerprint of the molecular progression from DNA damage to apoptosis delineated by proteomics, while the p53 status remains the decisive factor determining which stress axis dominates in each cell line. In this way, it complements proteomics data and underscores its potential as a diagnostic tool for rapidly resolving treatment-induced molecular stress signatures in Hodgkin lymphoma cells. While this work was deliberately designed to investigate TP53-dependent differences within Hodgkin lymphoma cell lines, future comparative studies integrating SERS and MS-based proteomics across multiple cancer entities will be required to disentangle apoptosis-associated signatures from disease-specific molecular patterns.

Although patient-derived samples were not included, this study establishes a transferable methodological framework for translational research. The proteomic-spectroscopic workflow enables systematic assessment of treatment responses and the identification of robust biomarker signatures. Compared with classical functional in vitro readouts (viability, proliferation, cell-cycle, or apoptosis/necrosis assays), Raman spectroscopy provides label-free, rapid, and single-cell resolved molecular phenotyping, offering mechanistic insight beyond endpoint-based measurements.

Future work will refine analytical protocols and expand the workflow toward full automated multi-omics integration by combining SERS with proteomics, transcriptomics, and metabolomics. Untargeted proteomics and independent molecular-biological validation assays will further substantiate the multi-omics findings. This strategy is expected to sharpen biomarker detection, elucidate underlying mechanisms, and enhance the interpretability of complex molecular responses in hematologic cancers, with potential applicability to additional indications such as metastatic melanoma, melanocytes, and patient-derived leukemia cells, ultimately supporting accelerated clinical translation of molecular stress phenotyping.

## Supplementary Information

Below is the link to the electronic supplementary material.

Supplementary file1 (DOCX 507 KB)

## Data Availability

The whole code for data analysis is available on GitHub (https://github.com/davidlilek/ABC_ANAKON_2025). Data are available upon reasonable request.

## Footnotes

1 https://github.com/davidlilek/ABC_ANAKON_2025/tree/master/sers/results_machinelearning

2 https://github.com/davidlilek/ABC_ANAKON_2025/tree/master/proteomics

## References

[1] International Agency for Research on Cancer. Global Cancer Observatory. https://gco.iarc.fr/. Accessed 15 Dec 2025.

[2] Kishida M, Fujisawa M, Steidl C. Molecular biomarkers in classic Hodgkin lymphoma. Semin Hematol. 2024;61:221–8. 10.1053/j.seminhematol.2024.05.005. 38969539 10.1053/j.seminhematol.2024.05.005

[3] Mottok A, Steidl C. Biology of classical Hodgkin lymphoma: implications for prognosis and novel therapies. Blood. 2018;131:1654–65. 10.1182/blood-2017-09-772632. 29500175 10.1182/blood-2017-09-772632

[4] Munir F, Hardit V, Sheikh IN, AlQahtani S, He J, Cuglievan B, et al. Classical Hodgkin lymphoma: from past to future—a comprehensive review of pathophysiology and therapeutic advances. Int J Mol Sci. 2023;24(12): 10095. 10.3390/ijms241210095. 37373245 10.3390/ijms241210095 PMC10298672

[5] Dhillon A, Singh A, Bhalla VK. A systematic review on biomarker identification for cancer diagnosis and prognosis in multi-omics: from computational needs to machine learning and deep learning. Arch Comput Methods Eng. 2023;30:917–49. 10.1007/s11831-022-09821-9.

[6] Quezada H, Guzmán-Ortiz AL, Díaz-Sánchez H, Valle-Rios R, Aguirre-Hernández J. Omics-based biomarkers: current status and potential use in the clinic. Boletín Médico Del Hospital Infantil de México (English Edition). 2017;74:219–26. 10.1016/j.bmhime.2017.11.030. 10.1016/j.bmhimx.2017.03.003 29382490

[7] Rai V, Mukherjee R, Ghosh AK, Routray A, Chakraborty C. Omics” in oral cancer: new approaches for biomarker discovery. Arch Oral Biol. 2018;87:15–34. 10.1016/j.archoralbio.2017.12.003. 29247855 10.1016/j.archoralbio.2017.12.003

[8] Hristova VA, Chan DW. Cancer biomarker discovery and translation: proteomics and beyond. Expert Rev Proteomics. 2019;16:93–103. 10.1080/14789450.2019.1559062. 30556752 10.1080/14789450.2019.1559062 PMC6635916

[9] Wu L, Qu X. Cancer biomarker detection: recent achievements and challenges. Chem Soc Rev. 2015;44(10):2963–97. 10.1039/C4CS00370E. 25739971 10.1039/c4cs00370e

[10] Xiao Y, Bi M, Guo H, Li M. Multi-omics approaches for biomarker discovery in early ovarian cancer diagnosis. EBioMedicine. 2022;79: 104001. 10.1016/j.ebiom.2022.104001. 35439677 10.1016/j.ebiom.2022.104001 PMC9035645

[11] Turanli B, Yildirim E, Gulfidan G, Arga KY, Sinha R. Current state of “Omics” biomarkers in pancreatic cancer. J Pers Med. 2021;11: 127. 10.3390/jpm11020127. 33672926 10.3390/jpm11020127 PMC7918884

[12] Zhang Y, Zhao S, Zheng J, He L. Surface-enhanced Raman spectroscopy (SERS) combined techniques for high-performance detection and characterization. TrAC Trends Anal Chem. 2017;90:1–13. 10.1016/j.trac.2017.02.006.

[13] Auner GW, Koya SK, Huang C, Broadbent B, Trexler M, Auner Z, et al. Applications of raman spectroscopy in cancer diagnosis. Cancer Metastasis Rev. 2018;37:691–717. 10.1007/s10555-018-9770-9. 30569241 10.1007/s10555-018-9770-9 PMC6514064

[14] Cialla-May D, Bonifacio A, Bocklitz T, Markin A, Markina N, Fornasaro S, et al. Biomedical SERS – the current state and future trends. Chem Soc Rev. 2024;53(18):8957–79. 10.1039/D4CS00090K. 39109571 10.1039/d4cs00090k

[15] Cialla-May D, Zheng XS, Weber K, Popp J. Recent progress in surface-enhanced Raman spectroscopy for biological and biomedical applications: from cells to clinics. Chem Soc Rev. 2017;46:3945–61 DOI as in original. 28639667 10.1039/c7cs00172j

[16] Barucci A, D’Andrea C, Farnesi E, Banchelli M, Amicucci C, De Angelis M, et al. Label-free SERS detection of proteins based on machine learning classification of chemo-structural determinants. Analyst. 2021;146:674–82. 10.1039/D0AN02137G. 33210104 10.1039/d0an02137g

[17] Vázquez-Iglesias L, Stanfoca Casagrande GM, García-Lojo D, Ferro Leal L, Ngo TA, Pérez-Juste J, et al. SERS sensing for cancer biomarker: approaches and directions. Bioact Mater. 2024;34:248–68. 10.1016/j.bioactmat.2023.12.018. 38260819 10.1016/j.bioactmat.2023.12.018 PMC10801148

[18] Zimmermann D. Surface-enhanced Raman spectroscopy for the detection of oncometabolites. Master Thesis, FH Wiener Neustadt, Biotech Campus Tulln; 2021.

[19] Blake N, Gaifulina R, Griffin LD, Bell IM, Thomas GMH. Machine learning of Raman spectroscopy data for classifying cancers: a review of the recent literature. Diagnostics. 2022;12: 1491. 10.3390/diagnostics12061491. 35741300 10.3390/diagnostics12061491 PMC9222091

[20] Ralbovsky NM, Lednev IK. Towards development of a novel universal medical diagnostic method: Raman spectroscopy and machine learning. Chem Soc Rev. 2020;49:7428–53. 10.1039/D0CS01019G. 32996518 10.1039/d0cs01019g

[21] De Luca AC, Reader-Harris P, Mazilu M, Mariggiò S, Corda D, Di Falco A. Reproducible surface-enhanced raman quantification of biomarkers in multicomponent mixtures. ACS Nano. 2014;8:2575–83. 10.1021/nn406200y. 24524333 10.1021/nn406200y

[22] Cutshaw G, Uthaman S, Hassan N, Kothadiya S, Wen X, Bardhan R. The emerging role of Raman spectroscopy as an omics approach for metabolic profiling and biomarker detection toward precision medicine. Chem Rev. 2023;123:8297–346. 10.1021/acs.chemrev.2c00897. 37318957 10.1021/acs.chemrev.2c00897 PMC10626597

[23] Dev K, Ho CJH, Bi R, Yew YW, S DU, Attia ABE, et al. Machine learning assisted handheld confocal Raman micro-spectroscopy for identification of clinically relevant atopic eczema biomarkers. Sensors. 2022;22:4674. 10.3390/s22134674. 10.3390/s22134674 PMC9269422 35808168

[24] Byrne HJ. Spectralomics – towards a holistic adaptation of label free spectroscopy. Vib Spectrosc. 2024;132: 103671. 10.1016/j.vibspec.2024.103671.

[25] Yang Y, Wu S, Chen Y, Ju H. Surface-enhanced Raman scattering sensing for detection and mapping of key cellular biomarkers. Chem Sci. 2023;14:12869–82. 10.1039/D3SC04650H. 38023499 10.1039/d3sc04650h PMC10664603

[26] Mohamed E, García Martínez DJ, Hosseini M-S, Yoong SQ, Fletcher D, Hart S, et al. Identification of biomarkers for the early detection of non-small cell lung cancer: a systematic review and meta-analysis. Carcinogenesis. 2024;45:1–22. 10.1093/carcin/bgad091. 38066655 10.1093/carcin/bgad091

[27] Chen X, Li X, Yang H, Xie J, Liu A. Diagnosis and staging of diffuse large B-cell lymphoma using label-free surface-enhanced Raman spectroscopy. Spectrochim Acta A Mol Biomol Spectrosc. 2022;267: 120571. 10.1016/j.saa.2021.120571. 34752994 10.1016/j.saa.2021.120571

[28] Cao H, Wu X, Shi H, Chu B, He Y, Wang H, Dong F. AI-assisted SERS imaging method for label-free and rapid discrimination of clinical lymphoma. J Nanobiotechnology. 2025;23: 295. 10.1186/s12951-025-03339-5. 40241186 10.1186/s12951-025-03339-5 PMC12001690

[29] Zimmermann D, Lilek D, Posch N, Hermann D-R, Pytel N, Herbinger B, Prohaska K. Classification of single cells by Raman spectroscopy and machine learning: comparison of common algorithms. Scientific Computing. Graz, Austria. 2023. pp. 12–19. https://2023.scientific-computing-conference.fh-joanneum.at/wp-content/uploads/sites/2/2023/04/Conference-Proceedings-Scientific-Computing.

[30] Zong C, Xu M, Xu LJ, Wei T, Ma X, Zheng XS, et al. Surface-enhanced Raman spectroscopy for bioanalysis: reliability and challenges. Chem Rev. 2018;118:4946–80. 10.1021/acs.chemrev.8b00026(doishortenedinoriginallist). 10.1021/acs.chemrev.7b00668 29638112

[31] Sloan-Dennison S, Wallace GQ, Hassanain WA, Laing S, Faulds K, Graham D. Advancing SERS as a quantitative technique: challenges, considerations, and correlative approaches to aid validation. Nano Converg. 2024;11: 33. 10.1186/s40580-024-00443-4. 39154073 10.1186/s40580-024-00443-4 PMC11330436

[32] Guo S, Popp J, Bocklitz T. Chemometric analysis in Raman spectroscopy from experimental design to machine learning–based modeling. Nat Protoc. 2021;16:5426–59. 10.1038/s41596-021-00620-3. 34741152 10.1038/s41596-021-00620-3

[33] Jiang Y, Rex DAB, Schuster D, Neely BA, Rosano GL, Volkmar N, et al. Comprehensive overview of bottom-up proteomics using mass spectrometry. ACS Meas Sci Au. 2024;4:338–417. 10.1021/acsmeasuresciau.3c00068. 39193565 10.1021/acsmeasuresciau.3c00068 PMC11348894

[34] Repetto O, De Re V, Mussolin L, Tedeschi M, Elia C, Bianchi M, et al. Proteomic profiles and biological processes of relapsed vs. non-relapsed pediatric Hodgkin lymphoma. Int J Mol Sci. 2020;21: 2185. 10.3390/ijms21062185. 32235718 10.3390/ijms21062185 PMC7139997

[35] Rudolf-Scholik J, Lilek D, Maier M, Reischenböck T, Maisl C, Allram J, Herbinger B, Rechthaler J. Increasing protein identifications in bottom-up proteomics of *T. castaneum* − exploiting synergies of protein biochemistry and bioinformatics. J Chromatogr B. 2024;1240: 124128. 10.1016/j.jchromb.2024.124128. 10.1016/j.jchromb.2024.124128 38759531

[36] Dupree EJ, Jayathirtha M, Yorkey H, Mihasan M, Petre BA, Darie CC. A critical review of bottom-up proteomics: the good, the bad, and the future of this field. Proteomes. 2020;8: 14. 10.3390/proteomes8030014. 32640657 10.3390/proteomes8030014 PMC7564415

[37] Duong V-A, Lee H. Bottom-up proteomics: advancements in sample preparation. Int J Mol Sci. 2023;24(6): 5350. 10.3390/ijms24065350. 36982423 10.3390/ijms24065350 PMC10049050

[38] Fornecker L-M, Muller L, Bertrand F, Paul N, Pichot A, Herbrecht R, et al. Multi-omics dataset to decipher the complexity of drug resistance in diffuse large B-cell lymphoma. Sci Rep. 2019;9: 895. 10.1038/s41598-018-37273-4. 30696890 10.1038/s41598-018-37273-4 PMC6351558

[39] Olivier M, Asmis R, Hawkins GA, Howard TD, Cox LA. The need for multi-omics biomarker signatures in precision medicine. Int J Mol Sci. 2019;20(19): 4781. 10.3390/ijms20194781. 31561483 10.3390/ijms20194781 PMC6801754

[40] Subramanian I, Verma S, Kumar S, Jere A, Anamika K. Multi-omics data integration, interpretation, and its application. Bioinform Biol Insights. 2020;14:10.1177/1177932219899051. 10.1177/1177932219899051 PMC7003173 32076369

[41] Tyanova S, Temu T, Cox J. The MaxQuant computational platform for mass spectrometry-based shotgun proteomics. Nat Protoc. 2016;11:2301–19. 10.1038/nprot.2016.136. 27809316 10.1038/nprot.2016.136

[42] Välikangas T, Suomi T, Elo LL. A comprehensive evaluation of popular proteomics software workflows for label-free proteome quantification and imputation. Brief Bioinform. 2018;19:1344–55. 10.1093/bib/bbx120. 28575146 10.1093/bib/bbx054 PMC6291797

[43] Etourneau L, Fancello L, Wieczorek S, Varoquaux N, Burger T. A new take on missing value imputation for bottom-up label-free LC-MS/MS proteomics. bioRxiv. 2023. 10.1101/2023.11.09.566355.

[44] Ali N, Girnus S, Rösch P, Popp J, Bocklitz T. Sample-size planning for multivariate data: a Raman-spectroscopy-based example. Anal Chem. 2018;90:12485–92. 10.1021/acs.analchem.8b02167. 30272961 10.1021/acs.analchem.8b02167

[45] Lilek D, Zimmermann D, Steininger L, Musso M, Wilts BD, Gamsjaeger S, Hermann D, Wiesner C, Grünfelder A, Herbinger B, Prohaska K. Machine learning of Raman spectroscopic data: comparison of different validation strategies. J Raman Spectrosc. 2025;56:867–77. 10.1002/jrs.6842.

[46] Drexler HG, Pommerenke C, Eberth S, Nagel S. Hodgkin lymphoma cell lines: to separate the wheat from the chaff. Biol Chem. 2018;399:511–23. 10.1515/hsz-2017-0321. 29533902 10.1515/hsz-2017-0321

[47] Ren B, Kwah MX-Y, Liu C, Ma Z, Shanmugam MK, Ding L, et al. Resveratrol for cancer therapy: challenges and future perspectives. Cancer Lett. 2021;515:63–72. 10.1016/j.canlet.2021.05.001. 34052324 10.1016/j.canlet.2021.05.001

[48] Prohaska K, Zimmermann D, Lilek D, Grünfelder A, Herbinger B. Raman analytics to monitor influence of resveratrol on lymphoma cells. Fachhochschulenkonferenz. Villach, Austria. 2022. http://ffhoarep.fh-ooe.at/handle/123456789/1580.FFH2022-Full-Paper138Panel6.

[49] Hande KR. Etoposide: four decades of development of a topoisomerase II inhibitor. Eur J Cancer. 1998;34:1514–21. 10.1016/S0959-8049(98)00228-7. 9893622 10.1016/s0959-8049(98)00228-7

[50] Britto Hurtado R, Cortez-Valadez M, Ramírez-Rodríguez LP, Larios-Rodriguez E, Alvarez RAB, Rocha-Rocha O, et al. Instant synthesis of gold nanoparticles at room temperature and SERS applications. Phys Lett A. 2016;380:2658–63. 10.1016/j.physleta.2016.05.052.

[51] Abueg LAL, Afgan E, Allart O, Awan AH, Bacon WA, et al. The Galaxy platform for accessible, reproducible, and collaborative data analyses: 2024 update. Nucleic Acids Res. 2024;52:W83–94. 10.1093/nar/gkae410. 38769056 10.1093/nar/gkae410 PMC11223835

[52] Schessner JP, Voytik E, Bludau I. A practical guide to interpreting and generating bottom-up proteomics data visualizations. Proteomics. 2022;22:10.1002/pmic.202100103. 10.1002/pmic.202100103 35107884

[53] Peng H, Wang H, Kong W, Li J, Goh WWB. Optimizing differential expression analysis for proteomics data via high-performing rules and ensemble inference. Nat Commun. 2024;15: 3922. 10.1038/s41467-024-47899-w. 38724498 10.1038/s41467-024-47899-w PMC11082229

[54] Zhu Y, Orre LM, Zhou Tran Y, Mermelekas G, Johansson HJ, Malyutina A, et al. DEqMS: a method for accurate variance estimation in differential protein expression analysis. Mol Cell Proteomics. 2020;19:1047–57. 10.1074/mcp.TIR119.001646. 32205417 10.1074/mcp.TIR119.001646 PMC7261819

[55] Salehi F, Abbasi E, Hassibi B. The impact of regularization on high-dimensional logistic regression. arXiv. 2019. https://arxiv.org/abs/1906.03761. Accessed 15 Dec 2025.

[56] Lundberg SM, Erion G, Chen H, DeGrave A, Prutkin JM, Nair B, et al. From local explanations to global understanding with explainable AI for trees. Nat Mach Intell. 2020;2:56–67. 10.1038/s42256-019-0138-9. 32607472 10.1038/s42256-019-0138-9 PMC7326367

[57] Wu T, Hu E, Xu S, Chen M, Guo P, Dai Z, et al. ClusterProfiler 4.0: a universal enrichment tool for interpreting omics data. Innovation. 2021;2: 100141. 10.1016/j.xinn.2021.100141. 34557778 10.1016/j.xinn.2021.100141 PMC8454663

[58] Humphrey SJ, Karayel O, James DE, Mann M. High-throughput and high-sensitivity phosphoproteomics with the EasyPhos platform. Nat Protoc. 2018;13:1897–916. 10.1038/s41596-018-0014-9. 30190555 10.1038/s41596-018-0014-9

[59] Meier F, Geyer PE, Virreira Winter S, Cox J, Mann M. BoxCar acquisition method enables single-shot proteomics at a depth of 10,000 proteins in 100 minutes. Nat Methods. 2018;15:440–8. 10.1038/s41592-018-0003-5. 29735998 10.1038/s41592-018-0003-5

[60] Ju S, Singh MK, Han S, Ranbhise J, Ha J, Choe W, et al. Oxidative stress and cancer therapy: controlling cancer cells using reactive oxygen species. Int J Mol Sci. 2024. 10.3390/ijms252212387. 39596452 10.3390/ijms252212387 PMC11595237

[61] Velegzhaninov I, Ievlev V, Pylina Y, Shadrin D, Vakhrusheva O. Programming of cell resistance to genotoxic and oxidative stress. Biomedicines. 2018;6: 5. 10.3390/biomedicines6010005. 29301323 10.3390/biomedicines6010005 PMC5874662

[62] Winczura A, Czubaty A, Winczura K, Masłowska K, Nałęcz M, Dudzińska DA, et al. Lipid peroxidation product 4-hydroxy-2-nonenal modulates base excision repair in human cells. DNA Repair. 2014;22:1–11. 10.1016/j.dnarep.2014.06.002. 25083554 10.1016/j.dnarep.2014.06.002

[63] Liang X, Weng J, You Z, Wang Y, Wen J, Xia Z, Huang S, Luo P, Cheng Q. Oxidative stress in cancer: from tumor and microenvironment remodeling to therapeutic frontiers. Mol Cancer. 2025;24: 219. 10.1186/s12943-025-02375-x. 40847302 10.1186/s12943-025-02375-x PMC12372290

