# Supplementary material for: Potential of SERS and proteomics for biomarker detection in cancer cells
Source: Anal Bioanal Chem. 2026 Mar 27;418(16):5247–65. doi: 10.1007/s00216-026-06459-5 (PMC13424330; doi:10.1007/s00216-026-06459-5)
Supplement: Supplementary file 1 — Supplementary file1 (DOCX 507 KB) [file 216_2026_6459_MOESM1_ESM.docx]

Content

[Methodical details Nano-LC-ESI-MS/MS 2](#_Toc223349670)

[Parameters used in MaxQuant 4](#_Toc223349671)

[Design and characterization of Au-NPs 5](#_Toc223349672)

[Data analysis parameters for SERS 6](#_Toc223349673)

[Detailed results of the machine learning models 7](#_Toc223349674)

[Detailed results proteomics 8](#_Toc223349675)

[Band Assignment LogL1 Coefficients 10](#_Toc223349676)

[Results Proteomics 16](#_Toc223349677)

[Manufacturer overview 24](#_Toc223349678)

[Literature 27](#_Toc223349679)

# Methodical details Nano-LC-ESI-MS/MS

Table S 1: Parameters used for nano-LC-ESI-MS/MS measurements

| Parameter Nano-LC | Specification/Value | |
| --- | --- | --- |
| RSLC nanoLC System | Thermo Scientific™; U3000 Nano Flow RSLCnano SYS W/O DET; 5200.0355 | |
| Nano-pump module and column oven | NCS-3500RS | |
| Autosampler | WPS-3000TPL RS | |
| UV-Detector | VWD-3100 Detector with flow-through cell; 214 nm | |
| Column | Acclaim™ PepMap™ 100 C18 LC Columns;  75 μm I.D. x 50 cm; 2 μm; P/N 164942 | |
| Precolumn | μ-Precolumn 300 μm I.D. x 5 mm C18 PepMap^TM^ 100, 5 μm, 100 Å; P/N 160454 | |
| Mobile Phase – Loading pump | 0.5% trifluoroacetic acid | |
| Flow rate – Loading pump | 15 µL/min | |
| Mobile Phase – Nano pump | A: 0.1% formic acid  B: 80% acetonitrile incl. 0.08% formic acid | |
| Flow rate – Nano pump | 0.3 µL/min | |
| Injection volume | 1 µL (via µL-pickup) | |
| Column oven temperature | 45 °C | |
| Duration of method | 120 min | |
| Elution Gradient | 0-5 min 3% B; 5-81 min 3%-40 % B; 81-83 min 40%-95% B, 83-100 min 95% B; 100-105 min 95%-3% B; 105-120 min 3% B | |
| Parameter Nano-ESI |  | |
| Spray voltage (\|kV\|) | 2 | |
| Spray current (µA) | 0.1 | |
| Capillary temperature (°C) | 275 | |
| S-lens RF level | 50 | |
| Parameter Q Exactive Plus Mass Spectrometer |  | |
| ***General***  Polarity  Lock masses | Positive 15 most positive | |
| *Full MS*  Resolution  AGC target  Maximum IT  Scan range | 70000  1e6  50 ms  375 to 1500 m/z | |
| Parameter Q Exactive Plus Mass Spectrometer | |  |
| *dd Settings*  Minimum AGC target  Intensity threshold  Apex trigger  Charge exclusion  Peptide match  Exclude isotope  Dynamic exclusion | 8.00e3  1.6e5  -  Unassigned, 1, 7, 8, >8  Preferred  On  50.0 s | |

Cont. Table S1: Parameters used for nano-LC-ESI-MS/MS measurements

| Parameter Q Exactive Plus Mass Spectrometer |  |
| --- | --- |
| dd-MS^2^/dd-SIM  Resolution  AGC target  Maximum IT  Loop count  TopN  Isolation window  Fixed first mass  (N)CE/stepped | 17500  1.e5  50 ms  15  15  2.0 m/z  -  nce_27 |

# Parameters used in MaxQuant

Table S2: Parameters used for protein identification/quantification in MaxQuant v.2.0.3.0 – only the most important parameters are shown.

| \| **Category** \| **Parameter** \| **Value / Setting** \| \| --- \| --- \| --- \| \| **Input and database** \| Input file type \| thermo.raw \| \|  \| FASTA database \| User-selected \| \|  \| Identifier parse rule \| >([^\s]*) \| \|  \| Description parse rule \| >(.*) \| \| **Peptide identification** \| Minimum peptide length \| 7 \| \|  \| Maximum peptide mass (Da) \| 4600 \| \|  \| Minimum unique peptides \| 0 \| \|  \| Missed cleavages \| 2 \| \|  \| Enzyme \| Trypsin/P \| \|  \| Digestion mode \| Specific \| \| **Modifications** \| Fixed modifications \| Carbamidomethyl (C) \| \|  \| Variable modifications \| Oxidation (M), Acetyl (Protein N-term) \| \| **FDR control** \| PSM FDR \| 0.01 (1%) \| \|  \| Protein FDR \| 0.01 (1%) \| \|  \| Decoy mode \| Revert \| \| **Quantification (LFQ)** \| Quantitation method \| Label-free quantification (LFQ) \| \|  \| Peptides for quantification \| Unique + razor \| \|  \| Use only unmodified peptides \| Yes \| \|  \| LFQ minimum ratio count \| 2 \| \|  \| LFQ minimum number of neighbours \| 3 \| \|  \| LFQ average number of neighbours \| 6 \| \|  \| Normalization type \| Classic \| \|  \| Separate LFQ in parameter groups \| Not selected \| \|  \| Stabilize large LFQ ratios \| Enabled \| \|  \| Require MS/MS for LFQ comparisons \| Enabled \| \| **Advanced options** \| iBAQ calculation \| No \| \|  \| Match between runs \| No \| \|  \| Include contaminants \| Yes \| \|  \| Calculate peak properties \| Disabled \| \| **Output** \| Generated files \| Protein Groups \| \|  \| PTXQC report \| True \| \| **Instrumental / search tolerances** \| First search peptide tolerance \| 20 ppm \| \| Main search peptide tolerance \| 4.5 ppm \| \| Isotope match tolerance \| 2 ppm \| |  |
| --- | --- | --- | --- | --- | --- | --- | --- | --- | --- | --- | --- | --- | --- | --- | --- | --- | --- | --- | --- | --- | --- | --- | --- | --- | --- | --- | --- | --- | --- | --- | --- | --- | --- | --- | --- | --- | --- | --- | --- | --- | --- | --- | --- | --- | --- | --- | --- | --- | --- | --- | --- | --- | --- | --- | --- | --- | --- | --- | --- | --- | --- | --- | --- | --- | --- | --- | --- | --- | --- | --- | --- | --- | --- | --- | --- | --- | --- | --- | --- | --- | --- | --- | --- | --- | --- | --- | --- | --- | --- | --- | --- | --- | --- | --- | --- | --- | --- | --- | --- | --- | --- | --- | --- | --- |
|  |  |

# Design and characterization of Au-NPs

Au-NPs prepared according to Britto Hurtado et al. [1] have previously been applied successfully in SERS-based biological studies [2]. For NP preparation, a 50 mM solution of ascorbic acid and a 100 mM solution of sucrose were used. The reduction solution was made by mixing 0.5 mL of both solutions and diluting the mixture to 10 mL with HPLC-grade water. A 1 mM HAuCl_4_ solution was prepared by diluting 1.5 mL of the HAuCl_4_ stock (20 mM chloroauric acid stock solution by dissolving 40 mg HAuCl_4_ 3H_2_O in 5 mL HPLC-grade water) to 30 mL with HPLC grade water. The reduction solution was then quickly added to the gold solution, resulting in a color change to red-purple. UV/Vis spectra of the NPs were acquired using a Tecan Plate Reader in the range of 380–750 nm. A characteristic surface plasmon resonance peak at ~500-550nm for gold indicated the successful preparation of SERS-active nanoparticles. Additionally, SEM images were generated with SEM Ultra Plus (Zeiss) at the PLUS University Salzburg to assess the nanoparticle size.

**(a)**

**(b)**


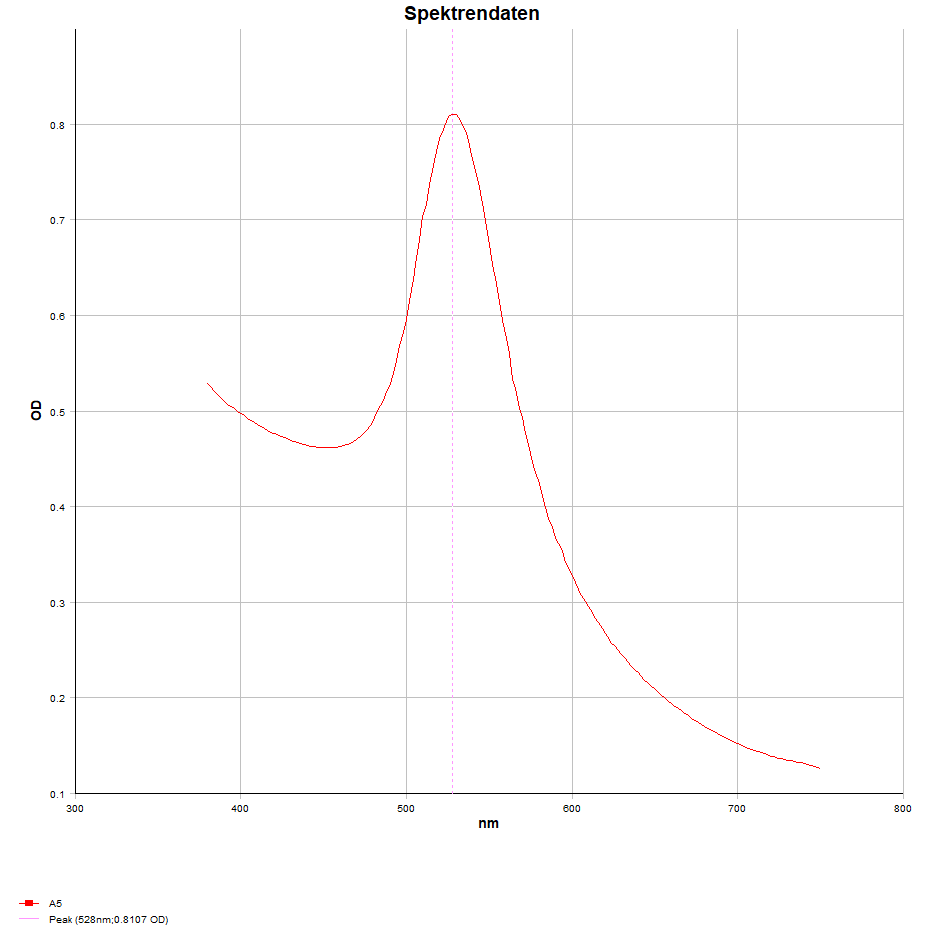




*Figure S1: (a) UV-Vis spectrum, x-axis wavelength from 380 to 750 nm, y-axis the intensity in au, the maximum at 528 nm indicating the nanoparticle size of around 40 nm. (b) SEM image of 40 nm sucrose / ascorbic acid Au-NPs.*

# Data analysis parameters for SERS

Table S 3: Parameters for the automated QC tool used and pre-processing for the different Raman datasets. The wavenumber was restricted to a region of interest. The spectral range used for each dataset is shown. For peak detection the window-size, threshold and minimum height for the calculation are shown. The scoring is based on the number of peaks as well as their heights (QC_SCORE and QC_PEAKS). The number of spectra kept after QC was set to 300 (QC_NUM).

|  | Settings |
| --- | --- |
| **Spectral Range** |  |
| Range low [cm^-1^] | 450 |
| Range high [cm^-1^] | 1650 |
| **Peak Detection** |  |
| QC_WINDOW | 35 |
| QC_TRESHOLD | 0.001 |
| QC_MIN_HEIGHT | 50 |
| **Scoring** |  |
| QC_SCORE | 1 |
| QC_PEAKS | 1 |
| QC_NUM | 300 |

Table S 4: Hyperparameters for each machine learning algorithm are specified with their minimum and maximum values including C representing regularization strength for L1 or L2 regularization; α as the complexity parameter for minimal cost-complexity pruning in the decision tree model and Feature Subsample referring to the fraction of randomly selected features used for each tree in a random forest model.

| Model | Parameter | Values |
| --- | --- | --- |
| PCA-LDA | Number of components | 1-50 |
| NMF-LDA | Number of components | 5-40 |
| FA-LDA | Number of components | 5-40 |
| Peak-Picking LDA | Minimum distance | 10-150 |
| Logistic Regression L1 | C | 10^-2^-10^1^ |
| Logistic Regression L2 | C | 10^-5^-10^-1^ |
| SVM L1 | C | 10^-3^-10 |
| SVM L2 | C | 10^-5^-10^-1^ |
| Decision Tree | α | 10^-3^-10^-1^ |
| Random Forest | Feature Subsample | 0.01-0.2 |
| GDBT | Learning Rate | 0.01-0.2 |

# Detailed results of the machine learning models

All machine learning models were evaluated using nested cross-validation, with hyperparameter optimization performed exclusively within the inner cross-validation loop. Model performance metrics, including accuracy, ROC curves, AUC values, F1 scores, and confusion matrices, were computed on the outer test folds only.

Hyperparameter search spaces were predefined and constrained to prevent overly complex model configurations. Detailed training and test results, quality control metrics, and full model diagnostics for all classifiers are provided in the accompanying reproducible HTML reports available in the GitHub repository - <https://github.com/davidlilek/ABC_ANAKON_2025/tree/master/sers/results_machinelearning>

# Detailed results proteomics

**Data and Code Availability**

Detailed proteomics workflows and results are publicly available in the GitHub repository, including RMarkdown files, rendered HTML outputs, and raw result tables (Excel format). PTXQC reports for each cell line are also provided to ensure transparency of data quality assessment.

Repository:

<https://github.com/davidlilek/ABC_ANAKON_2025/tree/master/proteomics>

The repository contains the analysis version used for this manuscript and includes:

- reproducibility.Rmd (analyses corresponding to Figures 3–4)
- heatmap_poi.Rmd (Figure 5)
- evaluation_reproducibility_rev001.Rmd (comprehensive reproducibility assessment as a supplement to reproducibility.Rmd)
- evaluation_diffproteomics_L428.Rmd
- evaluation_diffproteomics_L540_living.Rmd
- evaluation_diffproteomics_L540_cryo.Rmd
- proteomics_detailed_results.xlsx (complete differential expression results)
- PTXQC quality control reports for each cell line (ptxqc_*.pdf)

**Reproducibility and Biological Variability Assessment**

Additionally, to the main document the quality of the data was assessed using PTXQC in combination with established visualization strategies for bottom-up proteomics. Below, we provide a brief summary of the selected results. Further details, code and plots can be found in the "evaluation_reproducibility_rev001.Rmd" file.

**PCA** was performed on log2-transformed LFQ intensities after centering. For each cell line, PCA was first conducted separately for each treatment condition to assess within-condition variability and replicate clustering. Subsequently, a combined PCA including all treatment groups was performed for the respective cell line to evaluate treatment-driven separation and global variance structure. The same analytical strategy was applied consistently to all investigated cell lines. PCA revealed a primary separation according to treatment condition predominantly along PC1 and, to a lesser extent, along PC2, across all analyzed cell lines. Measurement replicates exhibited the most compact clustering pattern. Technical replicates grouped consistently within treatment conditions, whereas biological replicates showed slightly increased dispersion, as expected for biological variability. This pattern was particularly evident for L-540 living and L-540 cryo cells. In L-428 cells, a slightly increased dispersion of one replicate was observed, which is consistent with the corresponding CV and correlation analyses. No systematic clustering according to measurement batch or LC–MS run was detected.

Biological reproducibility was additionally evaluated using coefficient of variation (**CV**) calculations on non-log-transformed LFQ intensities per protein and per treatment group, as well as Pearson **correlation analysis** between biological replicates. For L-540 living cells median CV values ranged from 6.8% (Etoposide) to 17.7% (Resveratrol). Between 56% and 92% of proteins exhibited CV values below 20%, while only 2.7–23.3% exceeded 30% variability. Pearson correlation coefficients between biological replicates ranged from 0.953 to 0.988, indicating strong concordance across treatment conditions. For L-540 cryo cells median CV values ranged from 8.2% (RE) to 18.2% (Etoposide). The proportion of proteins with CV <20% ranged between 54% and 84%, whereas 4.6–24.9% exceeded 30%. Biological replicate correlations ranged from 0.962 to 0.985, confirming robust reproducibility. L-428 cells exhibited moderately higher biological variability compared to L-540 cells. Median CV values ranged from 19.6% to 21.9% across treatments, with approximately 45–50% of proteins showing CV <20% and around 31–33% above 30%. Pearson correlation coefficients ranged from 0.77 to 0.98. The increased dispersion was primarily driven by one biological replicate generated at a distinct cultivation timepoint, which exhibited a lower number of quantified proteins. Importantly, variability patterns were consistent across treatments, suggesting intrinsic biological heterogeneity rather than systematic technical bias.

This analysis demonstrates a high concordance between biological replicates in L-540 cell lines and a moderately increased, yet acceptable, level of biological variability in L-428 cells. Across all cell lines, variance was primarily driven by treatment effects rather than within-group or technical/measuring variability. Overall, these results support the robustness of the dataset and its suitability for downstream differential expression and biomarker analyses.

# Band Assignment LogL1 Coefficients

Table S 5: Band assignment of the coefficients. Intensity: Signals were grouped into very weak (< 0.05), weak (0.05–0.10), medium (0.10–0.30), strong (0.30–0.80), and very strong (> 0.80), with separate indication of positive and negative values. Comp.: Comparison: binary comparison of C(ontrol), E(toposide), R(esveratrol) and combined (RE) treatment. Band Assignment based on literature.

| **Intensity** | **Comp.** | **Cell line** | **Band Assignment (cm^-1^)** | **Literature** |
| --- | --- | --- | --- | --- |
| medium - | RE vs E | L-540 | Phenylalanine (451–456) | [3] |
| medium - | E vs R | L-428 | Phenylalanine (451–456) | [3] |
| medium + | RE vs C | L-428 | Phenylalanine (451–456) | [3] |
| strong + | E vs R | L-428 | (461) | - |
| weak - | RE vs C | L-428 | (474) | - |
| medium + | E vs R | L-540 | (476) | - |
| medium + | RE vs E | L-540 | Glycogen, DNA (481) | [4–6] |
| weak - | RE vs R | L-540 | Glycogen (489–490) | [4–7] |
| weak - | C vs R | L-540 | Glycogen (489–490) | [4–7] |
| weak - | C vs E | L-540 | Glycogen (489–490) | [4–7] |
| weak - | E vs R | L-540 | Amino Acids (496) | [4, 8] |
| medium + | E vs R | L-428 | Glycogen, DNA (514) | [6, 9, 10] |
| weak - | RE vs E | L-540 | Proteins, Phosphatidylinositol (520) | [3, 6] |
| weak - | RE vs C | L-428 | Proteins, Cholesterol (528–544) | [5, 6, 11] |
| weak + | RE vs E | L-540 | Proteins, Cholesterol (528–544) | [11, 12] |
| weak - | C vs R | L-428 | Proteins, Cholesterol (528–544) | [3, 6, 11] |
| medium - | E vs R | L-540 | (560) | - |
| medium + | E vs R | L-540 | (563) | - |
| medium + | RE vs R | L-540 | (563) | - |
| weak - | RE vs R | L-540 | Tryptophan, Cytosine, Guanine (572) | [5, 6, 13] |
| medium - | RE vs C | L-428 | Phosphatidylinositol (597–600) | [6] |
| strong - | E vs R | L-428 | Phosphatidylinositol (597–600) | [6] |
| medium - | E vs R | L-540 | (603) | - |
| medium + | E vs R | L-428 | (604) | - |
| medium + | C vs R | L-540 | Phenylalanine (610–634) | [6, 13, 14] |
| weak + | E vs R | L-540 | Phenylalanine (610–634) | [6, 13, 14] |
| weak + | RE vs C | L-428 | Phenylalanine (610–634) | [6, 13, 14] |
| weak - | RE vs R | L-428 | Phenylalanine (610–634) | [6, 13, 14] |
| medium - | RE vs E | L-540 | Phenylalanine (610–634) | [6, 13, 14] |
| medium + | RE vs E | L-540 | Phenylalanine (610–634) | [6, 13, 14] |
| medium - | RE vs C | L-428 | Phenylalanine, Cysteine, Tyrosine, Guanine, Thymine (637–649) | [3, 5, 6, 13] |
| medium + | C vs R | L-428 | Phenylalanine, Cysteine, Tyrosine, Guanine, Thymine (637–649) | [3, 5, 6, 13] |
| medium + | RE vs E | L-428 | Phenylalanine, Cysteine, Tyrosine, Guanine, Thymine (637–649) | [3, 5, 6, 13] |
| weak - | E vs R | L-540 | Phenylalanine, Cysteine, Tyrosine, Guanine, Thymine (637–649) | [3, 5, 6, 11] |
| medium + | RE vs C | L-428 | Cysteine, Tyrosine, Guanine, Thymine (661) | [6, 11] |
| medium + | C vs R | L-540 | Phenylalanine, Cysteine, Tyrosine, Guanine, Thymine (674–679) | [6, 9, 12, 15, 16] |

Cont. Table S 5: Band assignment of the LogL1 coefficients. Intensity: Signals were grouped into very weak (< 0.05), weak (0.05–0.10), medium (0.10–0.30), strong (0.30–0.80), and very strong (> 0.80), with separate indication of positive and negative values. Comp.: Comparison: binary comparison of C(ontrol), E(toposide), R(esveratrol) and combined (RE) treatment. Band Assignment based on literature.

| **Intensity** | **Comp.** | **Cell line** | **Band Assignment (cm^-1^)** | **Literature** |
| --- | --- | --- | --- | --- |
| strong - | RE vs C | L-428 | Phenylalanine, Cysteine, Tyrosine, Guanine, Thymine (674–679) | [6, 9, 12, 15, 16] |
| strong - | E vs R | L-428 | Phenylalanine, Tyrosine (683) | [14] |
| medium - | RE vs R | L-428 | Phenylalanine, Guanine (685) | [3, 14] |
| weak - | E vs R | L-540 | (692) | - |
| weak - | RE vs E | L-428 | (693) | - |
| medium + | RE vs E | L-540 | (694) | - |
| medium + | E vs R | L-428 | (695) | - |
| strong - | RE vs E | L-540 | Cholesterol, Cholesterol Ester (700) | [6] |
| strong + | E vs R | L-540 | Cholesterol, Cholesterol Ester (700) | [6] |
| weak + | C vs E | L-428 | Lipids, Phospholipids (715–716) | [3, 6, 9, 12, 14, 15] |
| medium + | C vs R | L-428 | Lipids, Phospholipids (715–716) | [3, 6, 9, 12, 14, 15] |
| strong - | C vs R | L-428 | Proteins, Cytochrome C, Adenine (730–740) | [5–7, 10, 14] |
| strong - | C vs E | L-428 | Proteins, Cytochrome C, Adenine (730–740) | [5–7, 10, 14] |
| strong - | C vs R | L-540 | Proteins, Cytochrome C, Adenine (730–740) | [5–7, 10, 14] |
| strong - | C vs E | L-540 | Proteins, Cytochrome C, Adenine (730–740) | [5–7, 10, 14] |
| strong + | RE vs C | L-540 | Proteins, Cytochrome C, Adenine (730–740) | [5–7, 10, 14] |
| strong + | RE vs C | L-428 | Proteins, Cytochrome C, Adenine (730–740) | [5–7, 10, 14] |
| medium - | E vs R | L-428 | Proteins, Cytochrome C, Adenine (730–740) | [5–7, 10, 14] |
| medium - | RE vs E | L-540 | Proteins, Cytochrome C, Adenine (730–740) | [5–7, 10, 14] |
| weak + | C vs E | L-540 | DNA, RNA (745) | [4, 6] |
| medium + | RE vs E | L-540 | DNA, Tryptophan, Cytochrome C (747–749) | [4–6, 14, 17] |
| strong + | E vs R | L-428 | DNA, Tryptophan, Cytochrome C (747–749) | [4–6, 14, 17] |
| medium + | E vs R | L-540 | DNA, Tryptophan, Cytochrome C (747–749) | [4–6, 14, 17] |
| medium - | RE vs C | L-428 | DNA, RNA, Phosphatidylinositol (776–777) | [5–7, 18] |
| medium - | RE vs R | L-540 | DNA, RNA, Phosphatidylinositol (776–777) | [5–7, 18] |
| medium - | E vs R | L-540 | DNA, RNA, Phosphatidylinositol (776–777) | [5–7, 18] |
| v. strong - | E vs R | L-428 | DNA, RNA (781–799) | [4, 7, 9, 10, 12, 14] |
| weak + | RE vs E | L-540 | DNA, RNA (781–799) | [4, 7, 9, 10, 12, 14] |
| medium + | E vs R | L-428 | DNA, RNA (781–799) | [4, 7, 9, 10, 12, 14] |
| medium - | E vs R | L-540 | Proline, (Poly)Saccharides (840–868) | [5, 6, 11, 13, 18] |
| medium + | RE vs E | L-540 | Proline, (Poly)Saccharides (840–868) | [5, 6, 11, 13, 18] |
| medium + | C vs R | L-428 | Proline, (Poly)Saccharides (840–868) | [5, 6, 11, 13, 18] |
| weak + | C vs E | L-428 | Proline, (Poly)Saccharides (840–868) | [5, 6, 11, 13, 18] |
| medium - | RE vs C | L-428 | Proline, (Poly)Saccharides (840–868) | [5, 6, 11, 13, 18] |
| medium - | E vs R | L-428 | Proline, (Poly)Saccharides (840–868) | [5, 6, 11, 13, 18] |
| medium + | E vs R | L-428 | Proline, (Poly)Saccharides (840–868) | [5, 6, 11, 13, 18] |
| weak - | RE vs E | L-428 | Saccharides (887–888) | [6] |
| medium - | RE vs E | L-540 | (905) | - |
| medium - | RE vs C | L-428 | (907) | - |
| weak - | C vs E | L-540 | (908) | - |
| weak + | C vs E | L-428 | Glucose? (910–913) | [6, 13] |
| medium - | E vs R | L-428 | Glucose? (910–913) | [6, 13] |

Cont. Table S 5: Band assignment of the LogL1 coefficients. Intensity: Signals were grouped into very weak (< 0.05), weak (0.05–0.10), medium (0.10–0.30), strong (0.30–0.80), and very strong (> 0.80), with separate indication of positive and negative values. Comp.: Comparison: binary comparison of C(ontrol), E(toposide), R(esveratrol) and combined (RE) treatment. Band Assignment based on literature.

| **Intensity** | **Comp.** | **Cell line** | **Band Assignment (cm^-1^)** | **Literature** |
| --- | --- | --- | --- | --- |
| weak + | RE vs E | L-540 | Glucose? (910–913) | [6, 13] |
| weak - | E vs R | L-540 | Glucose? (910–913) | [6, 13] |
| weak + | C vs R | L-428 | Proline, Glucose (917–918) | [5, 6, 18] |
| medium + | E vs R | L-540 | Proline, Glucose (917–918) | [5, 6, 18] |
| medium + | E vs R | L-428 | Proline, Valine (Amino Acids) (931) | [5–7, 9] |
| weak - | RE vs C | L-428 | Proteins, Phenylalanine (951) | [6, 18, 19] |
| weak - | C vs E | L-428 | Lipids, Phosphate (960–968) | [5, 6, 11, 12, 12] |
| weak - | C vs E | L-540 | Lipids, Phosphate (960–968) | [5, 6, 11, 12, 12] |
| weak - | RE vs E | L-428 | Lipids, Phosphate (960–968) | [5, 6, 11, 12, 12] |
| weak + | RE vs R | L-540 | Lipids, Phosphate (960–968) | [5, 6, 11, 12, 12] |
| medium + | C vs E | L-428 | Proteins, Lipids (977) | [3, 4, 6, 10, 18] |
| medium - | RE vs C | L-428 | Proteins (991) | [18] |
| medium + | C vs E | L-428 | Phenylalanine, Tryptophan (995–1007) | [3, 4, 6, 7, 11, 12, 15, 19] |
| strong + | C vs R | L-428 | Phenylalanine, Tryptophan (995–1007) | [3, 4, 6, 7, 11, 12, 15, 19] |
| very strong + | RE vs R | L-428 | Phenylalanine, Tryptophan (995–1007) | [3, 4, 6, 7, 11, 12, 15, 19] |
| very strong + | E vs R | L-428 | Phenylalanine, Tryptophan (995–1007) | [3, 4, 6, 7, 11, 12, 15, 19] |
| very strong + | RE vs E | L-428 | Phenylalanine, Tryptophan (995–1007) | [3, 4, 6, 7, 11, 12, 15, 19] |
| very strong + | RE vs C | L-428 | Phenylalanine, Tryptophan (995–1007) | [3, 4, 6, 7, 11, 12, 15, 19] |
| strong + | RE vs E | L-540 | Phenylalanine, Tryptophan (995–1007) | [3, 4, 6, 7, 11, 12, 15, 19] |
| medium - | E vs R | L-540 | Phenylalanine, Tryptophan (995–1007) | [3, 4, 6, 7, 11, 12, 15, 19] |
| strong - | E vs R | L-428 | Phenylalanine, Tryptophan (995–1007) | [3, 4, 6, 7, 11, 12, 15, 19] |
| strong - | RE vs E | L-428 | Phenylalanine, Tryptophan (995–1007) | [3, 4, 6, 7, 11, 12, 15, 19] |
| strong - | RE vs R | L-428 | Phenylalanine, Tryptophan (995–1007) | [3, 4, 6, 7, 11, 12, 15, 19] |
| medium - | RE vs E | L-540 | Phenylalanine, Tryptophan, Serine (1012–1013) | [11, 18] |
| weak + | E vs R | L-540 | Phenylalanine, Tryptophan, Serine (1012–1013) | [11, 18] |
| weak - | C vs R | L-540 | Phenylalanine (1029) | [3, 4, 6, 12, 15, 18] |
| weak + | RE vs C | L-540 | Phenylalanine (1029) | [3, 4, 6, 12, 15, 18] |
| weak + | RE vs R | L-540 | Phenylalanine, Proline, Collagen (1033) | [3, 6, 7, 15, 18] |
| weak - | C vs E | L-540 | Proline (1039) | [18] |
| weak + | RE vs E | L-540 | Proteins, Lipids (1051–1067) | [5, 6, 10, 12] |

Cont. Table S 5: Band assignment of the LogL1 coefficients. Intensity: Signals were grouped into very weak (< 0.05), weak (0.05–0.10), medium (0.10–0.30), strong (0.30–0.80), and very strong (> 0.80), with separate indication of positive and negative values. Comp.: Comparison: binary comparison of C(ontrol), E(toposide), R(esveratrol) and combined (RE) treatment. Band Assignment based on literature.

| **Intensity** | **Comp.** | **Cell line** | **Band Assignment (cm^-1^)** | **Literature** |
| --- | --- | --- | --- | --- |
| weak + | C vs E | L-540 | Proteins, Lipids (1051–1067) | [5, 6, 10, 12] |
| medium - | E vs R | L-540 | Proteins, Lipids (1051–1067) | [5, 6, 10, 12] |
| medium + | E vs R | L-540 | Lipids/Phospholipids, DNA (1072–1077) | [3, 6, 11, 19] |
| strong - | RE vs C | L-428 | Lipids/Phospholipids, DNA (1072–1077) | [3, 6, 11, 19] |
| strong - | E vs R | L-428 | Lipids/Phospholipids, DNA (1072–1077) | [3, 6, 11, 19] |
| medium - | RE vs R | L-540 | DNA, Proteins, Lipids/Phospholipids (1082–1104) | [3–5, 7, 9, 11, 12, 14, 15] |
| very strong + | E vs R | L-428 | DNA, Proteins, Lipids/Phospholipids (1082–1104) | [3–5, 7, 9, 11, 12, 14, 15] |
| weak + | RE vs E | L-428 | DNA, Proteins, Lipids/Phospholipids (1082–1104) | [3–5, 7, 9, 11, 12, 14, 15] |
| medium - | RE vs E | L-540 | DNA, Proteins, Lipids/Phospholipids (1082–1104) | [3–5, 7, 9, 11, 12, 14, 15] |
| weak - | C vs E | L-540 | DNA, Proteins, Lipids/Phospholipids (1082–1104) | [3–5, 7, 9, 11, 12, 14, 15] |
| weak - | RE vs E | L-428 | DNA, Proteins, Lipids/Phospholipids (1082–1104) | [3–5, 7, 9, 11, 12, 14, 15] |
| weak + | RE vs R | L-540 | Lipids, Proteins (1136–1139) | [7] |
| medium + | RE vs E | L-540 | Lipids, Proteins (1136–1139) | [7] |
| medium + | C vs E | L-540 | Lipids, Proteins (1136–1139) | [7] |
| medium - | RE vs E | L-540 | Glycogen (1147–1148) | [6] |
| weak + | E vs R | L-540 | Glycogen (1147–1148) | [6] |
| medium - | RE vs R | L-428 | Glycogen (1147–1148) | [6] |
| weak - | RE vs E | L-428 | Proteins, Glycogen (1149–1154) | [3–6, 14] |
| medium - | C vs R | L-428 | Proteins, Glycogen (1149–1154) | [3–6, 14] |
| weak - | C vs E | L-428 | Proteins, Glycogen (1149–1154) | [3–6, 14] |
| weak - | E vs R | L-540 | Amino Acids (1165) | [6] |
| medium + | E vs R | L-428 | Lipids, Amino Acids (1168) | [5, 6, 12] |
| weak - | RE vs C | L-428 | Tyrosine (1173) | [3, 5, 6, 9, 12] |
| medium - | E vs R | L-428 | DNA, Tyrosine (1176) | [5, 6, 9, 12, 14] |
| medium + | RE vs C | L-428 | DNA (1189) | [4, 5, 8, 20, 21] |
| medium + | RE vs E | L-540 | Amino Acids, DNA (1209) | [4–7, 12] |
| weak + | C vs E | L-428 | Amide III (Proteins), Phenylalanine, Tryptophan (1219–1255) | [5–7, 10, 12, 14, 15, 19] |
| medium - | RE vs E | L-540 | Amide III (Proteins), Phenylalanine, Tryptophan (1219–1255) | [5–7, 10, 12, 14, 15, 19] |
| medium - | RE vs C | L-428 | Amide III (Proteins), Phenylalanine, Tryptophan (1219–1255) | [5–7, 10, 12, 14, 15, 19] |
| medium - | C vs R | L-540 | Amide III (Proteins), Phenylalanine, Tryptophan (1219–1255) | [5–7, 10, 12, 14, 15, 19] |
| medium - | C vs E | L-540 | Amide III (Proteins), Phenylalanine, Tryptophan (1219–1255) | [5–7, 10, 12, 14, 15, 19] |
| medium + | RE vs C | L-428 | Amide III (Proteins), Phenylalanine, Tryptophan (1219–1255) | [5–7, 10, 12, 14, 15, 19] |

Cont. Table S 5: Band assignment of the LogL1 coefficients. Intensity: Signals were grouped into very weak (< 0.05), weak (0.05–0.10), medium (0.10–0.30), strong (0.30–0.80), and very strong (> 0.80), with separate indication of positive and negative values. Comp.: Comparison: binary comparison of C(ontrol), E(toposide), R(esveratrol) and combined (RE) treatment. Band Assignment based on literature.

| **Intensity** | **Comp.** | **Cell line** | **Band Assignment (cm^-1^)** | **Literature** |
| --- | --- | --- | --- | --- |
| weak + | RE vs E | L-428 | Amide III (Proteins), Phenylalanine, Tryptophan (1219–1255) | [5–7, 10, 12, 14, 15, 19] |
| weak + | E vs R | L-540 | Amide III (Proteins), Phenylalanine, Tryptophan (1219–1255) | [5–7, 10, 12, 14, 15, 19] |
| weak + | C vs R | L-428 | Amide III (Proteins), Phenylalanine, Tryptophan (1219–1255) | [5–7, 10, 12, 14, 15, 19] |
| weak - | E vs R | L-540 | Amide III (Proteins), Phenylalanine, Tryptophan (1219–1255) | [5–7, 10, 12, 14, 15, 19] |
| medium + | E vs R | L-428 | Amide III (Proteins, Lipids) (1260–1269) | [5–7, 11, 12, 14] |
| weak - | RE vs C | L-428 | Amide III (Proteins, Lipids) (1260–1269) | [5–7, 11, 12, 14] |
| medium + | C vs R | L-540 | Amide III (Proteins, Lipids) (1260–1269) | [5–7, 11, 12, 14] |
| strong + | C vs E | L-540 | Amide III (Proteins, Lipids) (1260–1269) | [5–7, 11, 12, 14] |
| strong - | RE vs C | L-540 | Amide III (Proteins, Lipids) (1260–1269) | [5–7, 11, 12, 14] |
| medium - | E vs R | L-428 | Amide III (Proteins, Lipids) (1260–1269) | [5–7, 11, 12, 14] |
| strong - | RE vs R | L-540 | Amide III (Proteins, Lipids) (1260–1269) | [5–7, 11, 12, 14] |
| strong - | RE vs E | L-540 | Amide III (Proteins, Phospholipids) (1270) | [3, 6, 9, 12] |
| medium + | RE vs E | L-540 | Amide III (Proteins) (1280) | [6, 15] |
| weak + | RE vs C | L-428 | Lipids, Cytosine (1291) | [6, 12, 14] |
| weak + | RE vs E | L-428 | Lipids, Cytosine (1291) | [6, 12, 14] |
| medium + | E vs R | L-540 | Lipids, Collagen (1304) | [5, 6, 10, 12, 14] |
| weak - | E vs R | L-540 | Lipids, Collagen, Cytochrome C (1309–1313) | [5, 10, 12, 17] |
| weak + | RE vs R | L-540 | Lipids, Collagen, Cytochrome C (1309–1313) | [5, 10, 12, 17] |
| medium - | E vs R | L-428 | DNA, Lipids, Proteins (1322) | [3, 6, 11, 12] |
| strong + | E vs R | L-428 | DNA, Lipids, Proteins, Collagen (1337–1341) | [3, 5, 6, 10–12, 19] |
| medium - | RE vs C | L-428 | DNA, Lipids, Proteins, Collagen (1337–1341) | [3, 5, 6, 10–12, 19] |
| medium + | RE vs C | L-428 | DNA, Lipids, Proteins (1364–1369) | [5, 6, 19] |
| weak + | RE vs E | L-428 | DNA, Lipids, Proteins (1364–1369) | [5, 6, 19] |
| medium + | RE vs R | L-428 | DNA, Lipids, Proteins (1364–1369) | [5, 6, 19] |
| weak - | RE vs E | L-540 | CH3 Bending Vibration (1385–1397) | [5, 6, 15] |
| medium + | RE vs E | L-540 | CH/CH2 Vibrations (1385–1397) | [6] |
| strong - | E vs R | L-428 | CH/CH2 Vibrations (1385–1397) | [6] |
| weak - | C vs R | L-540 | Proteins, Triglyceride, IgG? (1406–1411) | [3, 6] |
| weak + | E vs R | L-428 | Proteins, Triglyceride, IgG? (1406–1411) | [3, 6] |
| medium - | C vs R | L-428 | Proteins, Triglyceride, IgG? (1406–1411) | [3, 6] |
| weak + | RE vs C | L-428 | Proteins, Triglyceride, IgG? (1406–1411) | [3, 6] |
| medium - | E vs R | L-540 | 0 (1412) | - |
| medium - | RE vs E | L-540 | Adenine, Guanine, Deoxyribose? (1429) | [6, 14] |
| medium - | RE vs R | L-540 | Adenine, Guanine, Deoxyribose? (1429) | [6, 14] |
| weak + | RE vs E | L-428 | Lipids (1435) | [3, 5, 6] |
| medium + | RE vs E | L-540 | Carbohydrates, Lipids, Proteins (1441–1449) | [5–7, 9, 12, 19] |
| medium - | E vs R | L-428 | Carbohydrates, Lipids, Proteins (1441–1449) | [3–5, 9, 12, 14] |
| weak + | RE vs R | L-540 | Carbohydrates, Lipids, Proteins, Collagen (1462) | [5, 6, 8, 20, 22] |

Cont. Table S 5: Band assignment of the LogL1 coefficients. Intensity: Signals were grouped into very weak (< 0.05), weak (0.05–0.10), medium (0.10–0.30), strong (0.30–0.80), and very strong (> 0.80), with separate indication of positive and negative values. Comp.: Comparison: binary comparison of C(ontrol), E(toposide), R(esveratrol) and combined (RE) treatment. Band Assignment based on literature.

| **Intensity** | **Comp.** | **Cell line** | **Band Assignment (cm^-1^)** | **Literature** |
| --- | --- | --- | --- | --- |
| medium + | RE vs C | L-428 | Carbohydrates, Lipids/Collagen, Proteins (1462) | [3, 6, 8, 9, 14, 20, 22] |
| weak - | C vs E | L-540 | Serine, Proteins (1472) | [15, 18] |
| weak - | RE vs E | L-428 | Carbohydrates, Lipids/Collagen, Proteins, DNA (1484–1487) | [5, 6, 8, 20, 22] |
| medium + | RE vs E | L-540 | Carbohydrates, Lipids/Collagen, Proteins, DNA (1484–1487) | [3, 6, 8, 9, 14, 20, 22] |
| strong + | E vs R | L-428 | (1495) | - |
| strong - | RE vs E | L-540 | (1497) | - |
| strong + | E vs R | L-540 | (1497) | - |
| weak - | RE vs R | L-540 | Proteins (1502) | [3] |
| medium - | E vs R | L-540 | DNA (1509–1511) | [6] |
| medium - | E vs R | L-428 | DNA (1509–1511) | [6] |
| weak + | RE vs E | L-428 | DNA (1509–1511) | [6] |
| weak + | RE vs C | L-428 | (1528) | - |
| weak - | RE vs E | L-428 | (1540) | - |
| strong + | E vs R | L-428 | (1540) | - |
| medium + | C vs E | L-540 | Amide II (Protein), Tryptophan (1547) | [5, 6, 19] |
| weak - | RE vs E | L-540 | Tryptophan (1552–1553) | [5, 6, 13, 19] |
| medium - | E vs R | L-428 | Tryptophan (1552–1553) | [5, 6, 13, 19] |
| medium + | RE vs E | L-540 | Tryptophan, DNA (1565) | [3, 6] |
| medium - | RE vs C | L-428 | Lipids, DNA (1575) | [5, 6, 10, 12, 14] |
| medium + | E vs R | L-428 | Phenylalanine, Amide I (Proteins) (1601–1611) | [4, 6, 11, 12] |
| weak - | E vs R | L-428 | Amino Acids, Amide I (Proteins) (1601–1611) | [4, 6, 11, 12, 14] |
| strong - | RE vs E | L-540 | Tyrosine, Tryptophan, C=C; Proteins, Lipids/Collagen (1636–1637) | [5, 6, 10] |
| medium - | RE vs R | L-540 | Amide I (Protein, Collagen) (1636–1637) | [5, 6] |
| weak - | RE vs R | L-428 | Amide I (Protein, Collagen) (1636–1637) | [5, 6] |
| medium + | RE vs R | L-540 | Amide I (Protein) (1641–1646) | [5, 6, 12] |
| medium - | E vs R | L-540 | Amide I (Protein) (1641–1646) | [5, 6, 12] |
| strong + | RE vs E | L-540 | Amide I (Protein) (1641–1646) | [5, 6, 12] |
| weak + | C vs E | L-540 | Amide I (Protein) (1641–1646) | [5, 6, 12] |
| weak - | RE vs C | L-428 | Amide I (Protein) (1641–1646) | [5, 6, 12] |
| medium + | E vs R | L-540 | Amide I (Protein) (1641–1646) | [5, 6, 10, 12] |

# Results Proteomics

Table S 6: Gene annotation, pathway and protein function of proteins investigated in proteomics with references. Differential expressed proteins are defined with |log_2_FC| > 0.58 of binary comparisons; significant with adj.P < 0.10.

| **Gene** | **Pathway** | **Protein Function** | **Literature** |
| --- | --- | --- | --- |
| ACIN1 | Apoptosis | Apoptotic chromatin condensation inducer 1, activated by CASP3, in all conditions not differentially expressed, with no significance. | [23, 24] |
| AIFM1 | Apoptosis | Apoptosis-inducing factor, mitochondrion-associated 1, OxPhos, upstream activator of CASP3 | [25, 26] |
| APEX1 | DNA Damage, Chromatin Remodeling and Nuclear-Envelope Stress | Apurinic/apyrimidinic endonuclease, in base excision repair (BER) by cleaving damaged DNA, such as oxidative or alkylation damage. | [27, 28] |
| API5 | Apoptosis | Apoptosis Inhibitor 5, a key regulator in cell death and has functions related to cancer development, in all conditions not differentially expressed, but significant. | [29] |
| ARMT1 | DNA Damage, Chromatin Remodeling and Nuclear-Envelope Stress | Acidic residue methyltransferase, possible coupling with p53 and decision point between repair or apoptosis in replication stress. | [30] |
| BAG6 | Protein Misfolding, ER-Stress and Proteostasis | Multifunctional protein that acts as a chaperone, plays key roles in protein quality control, degradation, and biogenesis, in most conditions not differentially expressed, with no significance. | [31] |
| BANF1 | DNA Damage, Chromatin Remodeling and Nuclear-Envelope Stress | Barrier-to-Autointegration Factor 1 supports nuclear envelope (NE) reassembly after mitosis | [32] |
| BCCIP | DNA Damage, Chromatin Remodeling and Nuclear-Envelope Stress | BCCIP is a BRCA2-interacting protein involved in homologous recombination repair, p21 (CDKN1A)-mediated cell cycle control, and DNA replication fidelity. It coordinates p53/p21 signaling to arrest the cell cycle under genotoxic stress. | [33] |
| BCLAF1 | DNA Damage, Chromatin Remodeling and Nuclear-Envelope Stress | Nuclear transcriptional regulator BCLAF1 cooperates with p53/p21 and NF-κB to repress or activate stress-response genes, behaves nuclear transcriptional regulator largely pro-apoptotic under genotoxic stress, in all conditions not differentially expressed. | [34] |

Cont. Table S 6: Gene annotation, pathway and protein function of proteins investigated in proteomics with references. Differential expressed proteins are defined with |log2FC| > 0.58 of binary comparisons; significant with adj.P < 0.10.

| **Gene** | **Pathway** | **Protein Function** | **Literature** |
| --- | --- | --- | --- |
| CASP3 | Apoptosis | The executioner caspase in apoptosis. It cleaves key structural and repair proteins (PARP1, AIFM1, ACIN1, BANF1) and enforces the irreversible dismantling of nuclear and cytoskeletal architecture. | [35–37] |
| CCAR1 | Apoptosis | Cell Cycle and Apoptosis Regulator 1 (CCAR1), a p53 co-activator with pro-apoptotic and pro-checkpoint functions under genotoxic stress, was only upregulated by RE in L-540, while in all other conditions it was not differentially expressed (p-values 0.00 – 0.05). | [38] |
| CCAR2 | Apoptosis | Cell Cycle and Apoptosis Regulator 2, direct inhibitor of SIRT1, the NAD⁺-dependent deacetylase that suppresses p53, in all conditions not differentially expressed (p-values 0.00 – 0.06). | [38] |
| CD63 | Immune Modulation & Surface Remodeling | CD63 translocate to the plasma membrane, making it a widely used marker for basophil activation. | [39] |
| CD70 | Immune Modulation & Surface Remodeling | CD70 acts as a costimulatory molecule and plays an important role in the regulation of the immune system activation. | [40] |
| CDC37 | Protein Misfolding, ER-Stress and Proteostasis | Co-chaperone of HSP90, guiding proper folding and stability of protein kinases and signaling intermediates. | [41] |
| CDC42 | Protein Misfolding, ER-Stress and Proteostasis | Signal switch controlling actin, morphology, and motility, p53 can repress CDC42, overactive CDC42 promotes survival and invasion, in all conditions not differentially expressed (in L-540 cells p-values 0.00 – 0.07). | [42, 43] |
| CYC1 | Mitochondrial Dysfunction and Energy Collapse | Heme-containing component of mitochondrial complex III, changes in CYC1 expression are often a proxy for how the cytochrome c pool and electron-transport capacity behave during stress, slightly elevated under RE (+0,59) in L-540 cryo, in all other conditions not differentially expressed (p-values 0.00). | [44] |
| DDB1 | DNA Damage, Chromatin Remodeling and Nuclear-Envelope Stress | p53-dependent DNA-damage binding protein, in all conditions not differentially expressed (p-values 0.00 – 0.06). | [45] |
| DLST | Mitochondrial Dysfunction and Energy Collapse | Component of the rate-limiting TCA enzyme 2-oxoglutarate dehydrogenase | [46] |

Cont. Table S 6: Gene annotation, pathway and protein function of proteins investigated in proteomics with references. Differential expressed proteins are defined with |log2FC| > 0.58 of binary comparisons; significant with adj.P < 0.10.

| **Gene** | **Pathway** | **Protein Function** | **Literature** |
| --- | --- | --- | --- |
| EEF1E1 | DNA Damage, Chromatin Remodeling and Nuclear-Envelope Stress | Eukaryotic translation elongation factor 1 epsilon 1, under RE slightly differentially expressed in L-428 (RE +0,59), but in all other conditions not differentially expressed (p-values 0.00 – 0.05). | [43] |
| EPS15L1 | Apoptosis | Membrane-associated adaptor protein that participates in clathrin-mediated endocytosis - vesicle-regulatory aspect, in all conditions not differentially expressed, with no significance. | [47] |
| FEN1 | DNA Damage, Chromatin Remodeling and Nuclear-Envelope Stress | The endonuclease FEN1, essential for Okazaki-fragment processing and base-excision repair (BER), in all conditions not differentially expressed (p-values 0.01 – 0.08), expression at baseline, coinciding with the activation of APEX1 and BCCIP and the downregulation of CASP3. | [48] |
| GAR1 | DNA Damage, Chromatin Remodeling and Nuclear-Envelope Stress | A core component of the H/ACA small nucleolar ribonucleoprotein (snoRNP) complex, under stress, reduced GAR1 indicates suppressed ribosome production, nucleolar stress, and activation of p53-dependent checkpoints, in all conditions not differentially expressed (p-values 0.00 – 0.04). | [49] |
| GRB2 | Metabolic and Stress Signaling Regulation | A small adaptor protein that links activated receptor tyrosine kinases (RTKs) (e.g., EGFR, FGFR, MET) to downstream pathways, notably RAS–RAF–MEK–ERK and PI3K–AKT. It connects extracellular survival signals to mitogenic and anti-apoptotic responses. | [50] |
| HDGF | Immune Modulation & Surface Remodeling | Hepatoma-Derived Growth Factor: primarily as a nuclear transcriptional regulator and mitogenic factor, promoting cell proliferation, angiogenesis, and survival, is often overexpressed in tumors, where it correlates with poor prognosis, enhanced proliferation, and therapy resistance. | [51] |

Cont. Table S 6: Gene annotation, pathway and protein function of proteins investigated in proteomics with references. Differential expressed proteins are defined with |log2FC| > 0.58 of binary comparisons; significant with adj.P < 0.10.

| **Gene** | **Pathway** | **Protein Function** | **Literature** |
| --- | --- | --- | --- |
| HK1 /HK2 | Metabolic and Stress Signaling Regulation | Hexokinase 1 is constitutively expressed (“housekeeping” enzyme) and anchored tightly to the outer mitochondrial membrane via VDAC. It ensures basal glucose metabolism and directly couples glycolytic flux to mitochondrial ATP production. Hexokinase 2 is typically up-regulated by growth factors, hypoxia, or oncogenic signals (PI3K/AKT/mTOR). It localizes both in the cytosol and at mitochondria, where it can bind to VDAC and inhibit cytochrome c release, thus adding an anti-apoptotic function.  The two hexokinase isoforms, HK1 and HK2, cooperate to regulate glycolytic flux and mitochondrial integrity during stressHK2 isoform exchange links to the down-regulation of TCA and OXPHOS enzymes (SDHA/B, CYC1) and to VDAC remodeling, reflecting p53-mediated control of mitochondrial permeability. Together, HK1 and HK2 act as reciprocal sensors of metabolic stress, integrating p53 signaling with the decision between metabolic adaptation and mitochondrial apoptosis. HK2 was not differentially expressed (p-values 0.00 – 0.03). | [52] |
| KCNAB2 | Metabolic and Stress Signaling Regulation | Mitochondrial signaling: Potassium voltage-gated channel subfamily. A regulatory beta subunit 2 KCNAB2 integrates ion homeostasis with metabolic and apoptotic signaling - it showed biologically relevant regulation only in comparison to resveratrol monotherapy, while all other contrasts remained below the effect threshold. | [53] |
| LGALS1 | Apoptosis, Immune Modulation & Surface Remodeling | Metabolic stress: Galectin1 & Galectin3 are highly informative because they bridge metabolic stress, apoptosis, and immune modulation. | [54] |
| LGALS3 | Apoptosis, Immune Modulation & Surface Remodeling |  | [55, 56] |
| MANF | Protein Misfolding, ER-Stress and Proteostasis | Mesencephalic Astrocyte-Derived Neurotrophic Factor, is part of the unfolded protein response (UPR) machinery that protects cells from ER stress. | [57] |
| MYDGF | Protein Misfolding, ER-Stress and Proteostasis | Myeloid-derived growth factor (MYDGF) is a novel secreted protein with potent anti-apoptotic and tissue repairing properties, promotes tissue repair, cell survival, and anti-apoptotic signaling, especially under ischemic or oxidative stress, remaining in all conditions not differentially expressed (p-values 0.00 – 0.04). | [58] |

Cont. Table S 6: Gene annotation, pathway and protein function of proteins investigated in proteomics with references. Differential expressed proteins are defined with |log2FC| > 0.58 of binary comparisons; significant with adj.P < 0.10.

| **Gene** | | **Pathway** | **Protein Function** | **Literature** |
| --- | --- | --- | --- | --- |
| NENF | | Mitochondrial Dysfunction and Energy Collapse | Neudesin Neurotrophic Factor, NENF. In non-neuronal cells, NENF participates in adipogenesis, ER–mitochondrial signaling, and cell proliferation control via PI3K/AKT and MAPK pathway. | [59] |
| NFKB1 | | Metabolic and Stress Signaling Regulation | NF-κB p50 subunit is a transcription factor controlling genes for inflammation, stress response, survival, and immune modulation. In all conditions not differentially expressed (p-values 0.00 – 0.10). | [58] |
| NUDC | | Protein Misfolding, ER-Stress and Proteostasis | Secondary responder within the protein-misfolding and ER-stress network, p53 dependent, cytoskeletal and ER-stress linked factor. | [60–62] |
| NUDT5 | | Metabolic & Stress Signaling Regulation | Nudix Hydrolase 5 is a key metabolic and stress-response enzyme. | [63, 64] |
| NUP214 | | Metabolic & Stress Signaling Regulation | Binding partner of CRM1/exportin-1 and plays a role in p53 nuclear export, p21 localization, and overall checkpoint adaptation after DNA damage. When NUP214 is altered, nuclear signaling (including p53/p21 transport) becomes dysregulated. | [65] |
| OGDH | | Mitochondrial Dysfunction and Energy Collapse | 2-oxoglutarate dehydrogenase, the major rate-limiting point in the TCA cycle. | [66] |
| OGFR | | Metabolic & Stress Signaling Regulation | Opioid growth factor receptor, Zinc finger protein 2, regulates cell proliferation by up-regulating p21 (CDKN1A) and p16, leading to cell-cycle arrest in G1 phase. | [63, 67] |
| OXCT1 | | Mitochondrial Dysfunction and Energy Collapse | 3-oxoacid CoA-transferase 1 (OXCT1) is an enzyme that in humans is encoded by the OXCT1 gene. It is also known as succinyl-CoA-3-oxaloacid CoA transferase: slightly elevated in R treated L-540l iv cells (p-values 0.00 - 0.09). | [68] |
| PARP1 | | DNA Damage, Chromatin Remodeling and Nuclear-Envelope Stress | Poly ADP Ribose Polymerase1, PARP1, detects DNA strand breaks and uses NAD⁺ to synthesize poly-(ADP-ribose) chains on repair proteins, during apoptosis. PARP1 is cleaved by caspase-3, halting DNA repair and marking the irreversible transition from damage response to execution. | [69, 70] |
| PCYT1A | Protein Misfolding, ER-Stress and Proteostasis | | Phosphatidylcholine Cytidylyl Transferase A catalyzes the rate-limiting step of the CDP-choline (Kennedy) pathway, which produces phosphatidylcholine (PC), the main phospholipid of cell and mitochondrial membranes. | [71] |

Cont. Table S 6: Gene annotation, pathway and protein function of proteins investigated in proteomics with references. Differential expressed proteins are defined with |log2FC| > 0.58 of binary comparisons; significant with adj.P < 0.10.

| **Gene** | **Pathway** | **Protein Function** | **Literature** |
| --- | --- | --- | --- |
| PDCD4 | Apoptosis, Immune signaling modulation | Tumor suppressor and translation repressor, can promote apoptosis through p53-independent routes, often downstream of NF-κB or TGF-β signaling. Did not show any differential expression (p-values 0.00 – 0.01). | [72] |
| PDCD5 | Apoptosis | Pro-apoptotic cofactor that translocates from the cytoplasm to the nucleus early during apoptosis, interacts directly with p53, Tip60 (acetyltransferase), and AIFM1, an amplifier of the p53–caspase–AIFM1 signaling axis. | [73, 74] |
| PDCD6 | Protein Misfolding, ER-Stress and Proteostasis | Calcium sensors play a key role in processes such as endoplasmic reticulum (ER)-Golgi vesicular transport, endosomal biogenesis or membrane repair. | [75, 76] |
| PDCD6IP | Protein Misfolding, ER-Stress and Proteostasis | Programmed Cell Death 6 Interacting Protein, multifunctional scaffold protein that binds PDCD6. | [77] |
| PRCP | Apoptosis | Prolyl-Carboxypeptidase. In tumor or stress contexts, decreased PRCP often corresponds to oxidative load accumulation and impaired peptide turnover.  The lysosomal serine exopeptidase PRCP (Prolyl-Carboxypeptidase) modulates peptide turnover and oxidative-stress signaling by degrading angiotensin-II and related substrates. | [78] |
| PURA | Metabolic & Stress Signaling Regulation | [Purine-rich element-binding protein A, PURA, is a subtle but strategically important regulatory protein with strong mechanistic implications for DNA replication, transcription control, and stress response.](https://en.wikipedia.org/wiki/RNA_binding_protein) In all conditions not differentially expressed (p-values 0.00 – 0.01). | [79] |
| RBM4 | Metabolic & Stress Signaling Regulation | RNA Binding Motif Protein 4, a splicing regulator and RNA-binding protein that coordinates alternative splicing, mRNA translation, and cellular stress responses. | [80] |
| SDHA/B | Mitochondrial Dysfunction and Energy Collapse | Succinate dehydrogenase A/B, TCA cycle members | [81] |
| SERPINB6 | Protein Misfolding, ER-Stress and Proteostasis | Cytosolic serpin, protects cells from protease-mediated damage, stabilizes proteases during oxidative or inflammatory stress. | [82] |

Cont. Table S 6: Gene annotation, pathway and protein function of proteins investigated in proteomics with references. Differential expressed proteins are defined with |log2FC| > 0.58 of binary comparisons; significant with adj.P < 0.10.

| **Gene** | **Pathway** | **Protein Function** | **Literature** |
| --- | --- | --- | --- |
| SLC4A7 | Mitochondrial Dysfunction and Energy Collapse  Metabolic & Stress Signaling Regulation | Solute Carrier Family 4 Member 7, SLC4A7, is a membrane transporter that plays an important part in how cells handle pH balance, ion exchange, and energy metabolism. | [83, 84] |
| SMARCC2 | DNA Damage, Chromatin Remodeling and Nuclear-Envelope Stress | SMARCC2 is a core component of the SWI/SNF chromatin remodeling complex (BAF complex). BAF170, the SWI/SNF complex uses ATP hydrolysis to reposition nucleosomes, enabling or restricting access of transcription factors to DNA. In both cell models not differentially expressed (p-values 0.00 – 0.07). | [72] |
| SOD2 | Mitochondrial Dysfunction and Energy Collapse | Mitochondrial Superoxide Dismutase Mn-SOD | [85, 86] |
| ST13 | Protein Misfolding, ER-Stress and Proteostasis | An adaptor protein that interacts with heat shock proteins HSP70 and HSP90, acting as a co-chaperone to help them in protein folding and assembly processes. Significantly unchanged in all treatments. | [87] |
| STAT1 | Metabolic & Stress Signaling Regulation | Transducers and Activators of Transcription (STAT1–STAT6), cytoplasmic transcription factors that, once phosphorylated, dimerize and enter the nucleus to regulate gene expression, form a rapid signaling cascade that transduces extracellular cues (cytokines, growth factors, hormones) directly into transcriptional responses. | [88, 89] |
| STAT3 | Metabolic & Stress Signaling Regulation |  | [90] |
| STAT5A | Metabolic & Stress Signaling Regulation |  | [91] |
| STAT6 | Metabolic & Stress Signaling Regulation |  | [92] |
| SUCLG1/2 | Mitochondrial Dysfunction and Energy Collapse | The TCA cycle enzymes SUCLG1 and SUCLG2, which form the heterodimeric succinyl-CoA synthetase complex catalyzing substrate-level phosphorylation: Succinyl-CoA Synthetase Subunit Alpha, SUCLG1, and Succinyl-CoA Synthetase Subunit Beta, SUCLG2, are core mitochondrial enzymes in the TCA cycle, pattern of oxidative phosphorylation and energy-suppression. | [93] |

Cont. Table S 6: Gene annotation, pathway and protein function of proteins investigated in proteomics with references. Differential expressed proteins are defined with |log2FC| > 0.58 of binary comparisons; significant with adj.P < 0.10.

| **Gene** | **Pathway** | **Protein Function** | **Literature** |
| --- | --- | --- | --- |
| TNFAIP8 | Apoptosis, | Tumor Necrosis Factor Alpha–Induced Protein 8, is a cytoplasmic anti-apoptotic protein induced by TNF-α and NF-κB. | [94] |
| TNFRSF8 | Apoptosis, Immune Modulation & Surface Remodeling | Tumor Necrosis Factor Receptor Superfamily Member 8, CD30, is a membrane receptor of the TNF receptor superfamily, mainly expressed in Hodgkin/Reed–Sternberg cells. It can activate NF-κB, JNK, and PI3K/AKT pathways. | [95] |
| VDAC1 | Mitochondrial Dysfunction and Energy Collapse | Voltage-Dependent Anion Channel, VDAC family: VDAC1 is the major mitochondrial outer membrane pore, regulating exchange of ATP, ADP, metabolites, and ions between mitochondria and cytoplasm. It also serves as a gateway for cytochrome c release during apoptosis. VDAC2 binds and restrains BAK and BAX (pro-apoptotic factors), delaying mitochondrial outer membrane permeabilization (MOMP). VDAC3 is involved in mitochondrial redox balance, in somatic cells it regulates ROS signaling and protein import. | [96–98] |
| VDAC2 | Mitochondrial Dysfunction and Energy Collapse |  | [99] |
| VDAC3 | Mitochondrial Dysfunction and Energy Collapse |  | [99] |

Detailed results of the proteomics data can be found on GitHub. <https://github.com/davidlilek/ABC_ANAKON_2025/tree/master/proteomics>

# Manufacturer overview

The devices and substances used in this study are listed in Tab. S7-9 sorted by experiments.

Table S 7: Devices and substances used for cell culture including specifications.

| **Cell culture** | **Manufacturer** | **Specifications** |
| --- | --- | --- |
| **Devices** | | |
| Cell culture incubator | Heraeus | BBD6220 |
| Centrifuge | Eppendorf | 5415 D, 5415 R |
| Centrifuge | Rotina | 380 R |
| Filter tips | Sigma-Aldrich | Low Retention 1-200 µL, 1-1000 µL |
| Hemocytometer | Optik Labor | 0.1 mm depth,  0.0025 mm^2^ |
| Inverted microscope | Olympus | CKX41 |
| Lamina airflow | Thermo Scientific | MSC-Advantage |
| Light microscope | VWR | 630-1559 |
| Tissue culture flaks Standard | Sarstedt | T25 Art.-No: 83.3910.002 |
| **Substances** | | |
| Etoposide | Merck | ≥97 %, Art.-No. 341205 |
| RPMI-1640 cell culture medium | Sigma Aldrich | Sterile, Art.-No. R8758 |
| Fetal Calf Serum (FCS) | Sigma Aldrich | Sterile, Art.-No. F-7524 |
| HL cell line L-428 | DSMZ | ACC 197, Lot 19 |
| HL cell line L-540 | DSMZ | ACC 72, Lot 15 |
| Paraformaldehyde | Roth | Art.-No:0335.1 |
| Penicillin/Streptomycin | Sigma Aldrich | Sterile, Art.-No. P-4333 |
| Phosphate buffered saline buffer (DPBS) | Sigma Aldrich | Art.-No: D8537 |
| Resveratrol | Sigma-Aldrich | ≥99 %, Art.-No. R-5010 |
| Sodium Pyruvat solution | Sigma-Aldrich | Sterile; Art.-No. S8636 |
| Trypan blue | Fluka | Art.-No: 93590 |
| Trypsin/EDTA | Sigma-Aldrich | Art.-No: T2610 |

Table S 8: Devices and substances used for proteomics experiments including specifications.

| **Proteomics** | **Manufacturer** | **Specifications** |
| --- | --- | --- |
| **Devices** | | |
| Filter Tips | Biozym | SurPhob, Low Binding, sterile (20µL, 200µL) |
| Microcon Centrifugal Filters | Merck Millipore Ltd. | Regenerated Cellulose, 10000 NMWL, Art.-No.  2018-15386 |
| Nanodrop 1000 | PeqLab | G722 |
| Microplate Reader | Tecan | Infinite M Plex, 1811003729 |
| Protein LowBind Tube | Eppendorf | 0.5 mL, Art.-No:0030108434; 1.5 mL, Art.-No.  0030108.116 |
| Thermomixer comfort | Eppendorf | 5355-04279, Cyro |
| Ultrasonic bath | Bandelin Sonorex | RK510 |
| ZipTips | Merck | C18, P10  Art.-No: ZTC18S096 |
| **Substances** | | |
| Acetonitrile (HPLC grade) | Sigma-Aldrich | ≥99.9 %, Art.-No. 34851 |
| Albumin Standard (bovine Serum Albumin (BSA)) | Thermo Scientific | 2.0 mg/mL, Art.-No. 23209 |
| DL-Dithiothreitol (DTT) | Sigma-Aldrich | ≥99 %, Art.-No. D55455G |
| Formic acid (LC/MS grade) (FA) | Fisher chemical | ≥99 %, Art.-No. A117-50 |
| Halt^TM^Protease & Phosphatase Inhibitor Cocktail | Thermofisher | Art.-No: 78440 |
| Iodoacetamide (IAA) | Sigma-Aldrich | Art.-No. I1149-5G |
| Pierce^TM^BCA Protein Assay Kit | Thermo Scientific | Art.-No: 23225 |
| Pierce™ HeLa Protein Digest Standard, 20µg (quality sample) | Thermo Scientific | 20µg, Art.-No. 88328, Lot-No. XB343437 |
| Triethylammonium  Bicarbonate (TEAB) | Thermo Scientific | 1 M, 50 mL,  Art.-No.  90114 |
| Trifluoroacetic acid (TFA) | Merck | 25 %,  Art.-No.  CK1701051874 |
| Trypsin / Lys-C Mix | Promega | Art.-No. V5073 |
| Water HPLC Plus | Sigma-Aldrich | Art.-No: 7732-18-5 |

Table S 9: Devices and substances used for SERS experiments including specifications.

| **SERS** | **Manufacturer** | **Specifications** |
| --- | --- | --- |
| **Devices** | | |
| Calcium fluoride slides (CaF_2_) | Crystran unlimited | 76 x 26 x 1 x mm |
| Microspatula Teflon coat | VWR | 185 x 5 mm  Art.-No: 2312224 |
| Senterra I Confocal Raman Microscope (Raman) | Bruker | 532 nm (0.2-20 mW), 785 nm (1-100 mW);   20x, 50x, Olympus; CCD |
| **Substances** | | |
| Gold (III) chloride trihydrate | Sigma-Aldrich | Art.-No: 16961-25-4 |
| D-Sucrose | Fluka | > 99.5 %, Art.-No. 84099 |
| L-Ascorbic acid | Roth | ≥ 99 %, Art.-No. 6288.1 |
| Water HPLC Plus | Sigma-Aldrich | Art.-No: 7732-18-5 |

# Literature

1. Britto Hurtado R, Cortez-Valadez M, Ramírez-Rodríguez LP, Larios-Rodriguez E, Alvarez RAB, Rocha-Rocha O, Delgado-Beleño Y, Martinez-Nuñez CE, Arizpe-Chávez H, Hernández-Martínez AR, Flores-Acosta M (2016) Instant synthesis of gold nanoparticles at room temperature and SERS applications. Phys Lett A 380:2658–2663. https://doi.org/10.1016/j.physleta.2016.05.052

2. Zimmermann D, Lilek D, Posch N, Hermann D-R, Pytel N, Herbinger B, Prohaska K (2023) Classification of single cells by Raman spectroscopy and machine learning: comparison of common algorithms. Sci Comput 2023 Graz Austria

3. Veloso AB, Longo JPF, Muehlmann LA, Tollstadius BF, Souza PEN, Azevedo RB, Morais PC, Da Silva SW (2017) SERS Investigation of Cancer Cells Treated with PDT: Quantification of Cell Survival and Follow-up. Sci Rep 7:1–12. https://doi.org/10.1038/s41598-017-07469-1

4. Corsetti S, Rabl T, McGloin D, Nabi G (2018) Raman spectroscopy for accurately characterizing biomolecular changes in androgen-independent prostate cancer cells. J Biophotonics 11:e201700166. https://doi.org/10.1002/jbio.201700166

5. Stone N, Kendall C, Smith J, Crow P, Barr H (2004) Raman spectroscopy for identification of epithelial cancers. Faraday Discuss 126:141. https://doi.org/10.1039/b304992b

6. Movasaghi Z, Rehman S, Rehman IU (2007) Raman Spectroscopy of Biological Tissues. Appl Spectrosc Rev 42:493–541. https://doi.org/10.1080/05704920701551530

7. Martinelli LP, Iermak I, Moriyama LT, Requena MB, Pires L, Kurachi C (2020) Optical clearing agent increases effectiveness of photodynamic therapy in a mouse model of cutaneous melanoma: an analysis by Raman microspectroscopy. Biomed Opt Express 11:6516. https://doi.org/10.1364/BOE.405039

8. Kögler M, Itkonen J, Viitala T, Casteleijn MG (2020) Assessment of recombinant protein production in E. coli with Time-Gated Surface Enhanced Raman Spectroscopy (TG-SERS). Sci Rep 10:2472. https://doi.org/10.1038/s41598-020-59091-3

9. Neugebauer U, Clement JH, Bocklitz T, Krafft C, Popp J (2010) Identification and differentiation of single cells from peripheral blood by Raman spectroscopic imaging. J Biophotonics 3:579–587. https://doi.org/10.1002/jbio.201000020

10. Managò S, Valente C, Mirabelli P, De Luca AC (2015) Discrimination and classification of acute lymphoblastic leukemia cells by Raman spectroscopy. In: Optical Sensors 2015. International Society for Optics and Photonics, p 95060Z

11. Franco D, Trusso S, Fazio E, Allegra A, Musolino C, Speciale A, Cimino F, Saija A, Neri F, Nicolò MS, Guglielmino SPP (2017) Raman spectroscopy differentiates between sensitive and resistant multiple myeloma cell lines. Spectrochim Acta - Part Mol Biomol Spectrosc 187:15–22. https://doi.org/10.1016/j.saa.2017.06.020

12. Chen Y, Dai J, Zhou X, Liu Y, Zhang W, Peng G (2014) Raman Spectroscopy Analysis of the Biochemical Characteristics of Molecules Associated with the Malignant Transformation of Gastric Mucosa. PLoS ONE 9:e93906. https://doi.org/10.1371/journal.pone.0093906

13. De Gelder J, De Gussem K, Vandenabeele P, Moens L (2007) Reference database of Raman spectra of biological molecules. J Raman Spectrosc 38:1133–1147. https://doi.org/10.1002/jrs.1734

14. Brauchle E, Noor S, Holtorf E, Garbe C, Schenke-Layland K, Busch C (2014) Raman spectroscopy as an analytical tool for melanoma research. Clin Exp Dermatol 39:636–645. https://doi.org/10.1111/ced.12357

15. Zhu J, Zhou J, Guo J, Cai W, Liu B, Wang Z, Sun Z (2013) Surface-enhanced Raman spectroscopy investigation on human breast cancer cells. Chem Cent J 7:1–5. https://doi.org/10.1186/1752-153X-7-37

16. Madzharova F, Heiner Z, Gühlke M, Kneipp J (2016) Surface-Enhanced Hyper-Raman Spectra of Adenine, Guanine, Cytosine, Thymine, and Uracil. J Phys Chem C 120:15415–15423. https://doi.org/10.1021/acs.jpcc.6b02753

17. Pätzold R, Keuntje M, Theophile K, Müller J, Mielcarek E, Ngezahayo A, Anders-von Ahlften A (2008) In situ mapping of nitrifiers and anammox bacteria in microbial aggregates by means of confocal resonance Raman microscopy. J Microbiol Methods 72:241–248. https://doi.org/10.1016/j.mimet.2007.12.003

18. Draxler M (2024) Evaluating the effectiveness of in house made SERS substrate compared to commercial products for the identification of malignant melanoma. FH Wiener Neustadt, Biotech Campus Tulln

19. Rimskaya E, Shelygina S, Timurzieva A, Saraeva I, Perevedentseva E, Melnik N, Kudrin K, Reshetov D, Kudryashov S (2023) Multispectral Raman Differentiation of Malignant Skin Neoplasms In Vitro: Search for Specific Biomarkers and Optimal Wavelengths. Int J Mol Sci 24:14748. https://doi.org/10.3390/ijms241914748

20. Chisanga M, Muhamadali H, Ellis DI, Goodacre R (2018) Surface-Enhanced Raman Scattering (SERS) in Microbiology: Illumination and Enhancement of the Microbial World. Appl Spectrosc 72:987–1000. https://doi.org/10.1177/0003702818764672

21. Mosier-Boss P (2017) Review of SERS Substrates for Chemical Sensing. Nanomaterials 7:142. https://doi.org/10.3390/nano7060142

22. Strola SA, Baritaux J-C, Schultz E, Simon AC, Allier C, Espagnon I, Jary D, Dinten J-M (2014) Single bacteria identification by Raman spectroscopy. J Biomed Opt 19:111610. https://doi.org/10.1117/1.JBO.19.11.111610

23. Sahara S, Aoto M, Eguchi Y, Imamoto N, Yoneda Y, Tsujimoto Y (1999) Acinus is a caspase-3-activated protein required for apoptotic chromatin condensation. Nature 401:168–173. https://doi.org/10.1038/43678

24. Rigou P, Piddubnyak V, Faye A, Rain J-C, Michel L, Calvo F, Poyet J-L (2009) The antiapoptotic protein AAC-11 interacts with and regulates Acinus-mediated DNA fragmentation. EMBO J 28:1576–1588. https://doi.org/10.1038/emboj.2009.106

25. Susin SA, Lorenzo HK, Zamzami N, Marzo I, Snow BE, Brothers GM, Mangion J, Jacotot E, Costantini P, Loeffler M, Larochette N, Goodlett DR, Aebersold R, Siderovski DP, Penninger JM, Kroemer G (1999) Molecular characterization of mitochondrial apoptosis-inducing factor. Nature 397:441–446. https://doi.org/10.1038/17135

26. Candé C, Cohen I, Daugas E, Ravagnan L, Larochette N, Zamzami N, Kroemer G (2002) Apoptosis-inducing factor (AIF): a novel caspase-independent death effector released from mitochondria. Biochimie 84:215–222. https://doi.org/10.1016/S0300-9084(02)01374-3

27. Li M, Wilson DM 3rd (2014) Human apurinic/apyrimidinic endonuclease 1. Antioxid Redox Signal 20:678–707. https://doi.org/10.1089/ars.2013.5492

28. Kulaberoglu Y, Gundogdu R, Hergovich A (2016) The Role of p53/p21/p16 in DNA-Damage Signaling and DNA Repair. Elsevier Inc.

29. Abbas H, Derkaoui DK, Jeammet L, Adicéam E, Tiollier J, Sicard H, Braun T, Poyet J-L (2024) Apoptosis Inhibitor 5: A Multifaceted Regulator of Cell Fate. Biomolecules 14:. https://doi.org/10.3390/biom14010136

30. Perry JJP, Ballard GD, Albert AE, Dobrolecki LE, Malkas LH, Hoelz DJ (2015) Human C6orf211 Encodes Armt1, a Protein Carboxyl Methyltransferase that Targets PCNA and Is Linked to the DNA Damage Response. Cell Rep 10:1288–1296. https://doi.org/10.1016/j.celrep.2015.01.054

31. Krenciute G, Liu S, Yucer N, Shi Y, Ortiz P, Liu Q, Kim B-J, Odejimi AO, Leng M, Qin J, Wang Y (2013) Nuclear BAG6-UBL4A-GET4 Complex Mediates DNA Damage Signaling and Cell Death*. J Biol Chem 288:20547–20557. https://doi.org/10.1074/jbc.M112.443416

32. Burgess JT, Cheong CM, Suraweera A, Sobanski T, Beard S, Dave K, Rose M, Boucher D, Croft LV, Adams MN, O’Byrne K, Richard DJ, Bolderson E (2021) Barrier-to-autointegration-factor (Banf1) modulates DNA double-strand break repair pathway choice via regulation of DNA-dependent kinase (DNA-PK) activity. Nucleic Acids Res 49:3294–3307. https://doi.org/10.1093/nar/gkab110

33. Meng X, Fan J, Shen Z (2007) Roles of BCCIP in chromosome stability and cytokinesis. Oncogene 26:6253–6260. https://doi.org/10.1038/sj.onc.1210460

34. Repetto O, De Re V, Mussolin L, Tedeschi M, Elia C, Bianchi M, Buffardi S, Sala A, Burnelli R, Mascarin M (2020) Proteomic Profiles and Biological Processes of Relapsed vs. Non-Relapsed Pediatric Hodgkin Lymphoma. Int J Mol Sci 21:2185. https://doi.org/10.3390/ijms21062185

35. Dho SH, Cho M, Woo W, Jeong S, Kim LK (2025) Caspases as master regulators of programmed cell death: apoptosis, pyroptosis and beyond. Exp Mol Med 57:1121–1132. https://doi.org/10.1038/s12276-025-01470-9

36. Frazzi R, Valli R, Tamagnini I, Casali B, Latruffe N, Merli F (2013) Resveratrol-mediated apoptosis of hodgkin lymphoma cells involves SIRT1 inhibition and FOXO3a hyperacetylation. Int J Cancer 132:1013–1021. https://doi.org/10.1002/ijc.27748

37. Chehade H, Fox A, Mor GG, Alvero AB (2021) Determination of Caspase Activation by Western Blot. Methods Mol Biol Clifton NJ 2255:1–12. https://doi.org/10.1007/978-1-0716-1162-3_1

38. Johnson GS, Rajendran P, Dashwood RH (2020) *CCAR1* and *CCAR2* as gene chameleons with antagonistic duality: Preclinical, human translational, and mechanistic basis. Cancer Sci 111:3416–3425. https://doi.org/10.1111/cas.14579

39. Andreu Z, Yáñez-Mó M (2014) Tetraspanins in extracellular vesicle formation and function. Front Immunol 5:442. https://doi.org/10.3389/fimmu.2014.00442

40. Diegmann J, Junker K, Gerstmayer B, Bosio A, Hindermann W, Rosenhahn J, von Eggeling F (2005) Identification of CD70 as a diagnostic biomarker for clear cell renal cell carcinoma by gene expression profiling, real-time RT-PCR and immunohistochemistry. Eur J Cancer 41:1794–1801. https://doi.org/10.1016/j.ejca.2005.05.005

41. Taipale M, Jarosz DF, Lindquist S (2010) HSP90 at the hub of protein homeostasis: emerging mechanistic insights. Nat Rev Mol Cell Biol 11:515–528. https://doi.org/10.1038/nrm2918

42. Jordan SN, Canman JC (2012) Rho GTPases in animal cell cytokinesis: An occupation by the one percent. Cytoskeleton 69:919–930. https://doi.org/10.1002/cm.21071

43. Pitolli C, Wang Y, Candi E, Shi Y, Melino G, Amelio I (2019) P53-mediated tumor suppression: DNA-damage response and alternative mechanisms. Cancers 11:1–14. https://doi.org/10.3390/cancers11121983

44. Wallace DC (2012) Mitochondria and cancer. Nat Rev Cancer 12:685–698. https://doi.org/10.1038/nrc3365

45. Hu Z, Holzschuh J, Driever W (2015) Loss of DDB1 Leads to Transcriptional p53 Pathway Activation in Proliferating Cells, Cell Cycle Deregulation, and Apoptosis in Zebrafish Embryos. PLOS ONE 10:e0134299. https://doi.org/10.1371/journal.pone.0134299

46. Anderson NM, Qin X, Finan JM, Lam A, Athoe J, Missiaen R, Skuli N, Kennedy A, Saini AS, Tao T, Zhu S, Nissim I, Look AT, Qing G, Simon MC, Feng H (2021) Metabolic Enzyme DLST Promotes Tumor Aggression and Reveals a Vulnerability to OXPHOS Inhibition in High-Risk Neuroblastoma. Cancer Res 81:4417–4430. https://doi.org/10.1158/0008-5472.CAN-20-2153

47. Hawryluk MJ, Keyel PA, Mishra SK, Watkins SC, Heuser JE, Traub LM (2006) Epsin 1 is a Polyubiquitin‐Selective Clathrin‐Associated Sorting Protein. Traffic 7:262–281. https://doi.org/10.1111/j.1600-0854.2006.00383.x

48. Guo E, Ishii Y, Mueller J, Srivatsan A, Gahman T, Putnam CD, Wang JYJ, Kolodner RD (2020) FEN1 endonuclease as a therapeutic target for human cancers with defects in homologous recombination. Proc Natl Acad Sci 117:19415–19424. https://doi.org/10.1073/pnas.2009237117

49. Wang C, Meier UT (2004) Architecture and assembly of mammalian H/ACA small nucleolar and telomerase ribonucleoproteins. EMBO J 23:1857–1867. https://doi.org/10.1038/sj.emboj.7600181

50. Malagrinò F, Puglisi E, Pagano L, Travaglini-Allocatelli C, Toto A (2024) GRB2: A dynamic adaptor protein orchestrating cellular signaling in health and disease. Biochem Biophys Rep 39:101803. https://doi.org/10.1016/j.bbrep.2024.101803

51. Enomoto H, Nakamura H, Nishikawa H, Nishiguchi S, Iijima H (2020) Hepatoma-Derived Growth Factor: An Overview and Its Role as a Potential Therapeutic Target Molecule for Digestive Malignancies. Int J Mol Sci 21:. https://doi.org/10.3390/ijms21124216

52. He K, Tao F, Lu Y, Fang M, Huang H, Zhou Y (2025) The Role of HK2 in Tumorigenesis and Development: Potential for Targeted Therapy with Natural Products. Int J Med Sci 22:790–805. https://doi.org/10.7150/ijms.105553

53. Yee JX, Rastani A, Soden ME (2022) The potassium channel auxiliary subunit Kvβ2 ( *Kcnab2* ) regulates Kv1 channels and dopamine neuron firing. J Neurophysiol 128:62–72. https://doi.org/10.1152/jn.00194.2022

54. Griffiths A, Udomjarumanee P, Georgescu A-S, Barri M, Zinovkin DA, Pranjol MZI (2025) The Immunomodulatory Role of Galectin-1 in the Tumour Microenvironment and Strategies for Therapeutic Applications. Cancers 17:1888. https://doi.org/10.3390/cancers17111888

55. Jiang Q, Zhao Q, Li P (2025) Galectin-3 in metabolic disorders: mechanisms and therapeutic potential. Trends Mol Med 31:424–437. https://doi.org/10.1016/j.molmed.2024.11.006

56. Farhad M, Rolig AS, Redmond WL (2018) The role of Galectin-3 in modulating tumor growth and immunosuppression within the tumor microenvironment. Oncoimmunology 7:e1434467. https://doi.org/10.1080/2162402X.2018.1434467

57. Yu Y, Liu D, Chen X, Zhu L, Wan L (2021) MANF: A Novel Endoplasmic Reticulum Stress Response Protein—The Role in Neurological and Metabolic Disorders. Oxid Med Cell Longev 2021:6467679. https://doi.org/10.1155/2021/6467679

58. Ding Y, Xu X, Meng B, Wang L, Zhu B, Guo B, Zhang J, Xiang L, Dong J, Liu M, Xiang G (2023) Myeloid-derived growth factor alleviates non-alcoholic fatty liver disease alleviates in a manner involving IKKβ/NF-κB signaling. Cell Death Dis 14:376. https://doi.org/10.1038/s41419-023-05904-y

59. Ohta H, Kimura I, Konishi M, Itoh N (2015) Neudesin as a unique secreted protein with multi-functional roles in neural functions, energy metabolism, and tumorigenesis. Front Mol Biosci 2:. https://doi.org/10.3389/fmolb.2015.00024

60. Biebl MM, Delhommel F, Faust O, Zak KM, Agam G, Guo X, Mühlhofer M, Dahiya V, Hillebrand D, Popowicz GM, Kampmann M, Lamb DC, Rosenzweig R, Sattler M, Buchner J (2022) NudC guides client transfer between the Hsp40/70 and Hsp90 chaperone systems. Mol Cell 82:555-569.e7. https://doi.org/10.1016/j.molcel.2021.12.031

61. Garner MA, Gross AK (2025) NUDC Is Critical for Mitosis and Postmitotic Cell Maintenance Through Its Modulation of Dynein and Actin Cytoskeletal Reorganization. In: Bowes Rickman C, Grimm C, Anderson RE, Ash JD, Pierce E, Hollyfield JG (eds) Retinal Degenerative Diseases XX. Springer Nature Switzerland, Cham, pp 453–457

62. Dahiya V, Agam G, Lawatscheck J, Rutz DA, Lamb DC, Buchner J (2019) Coordinated Conformational Processing of the Tumor Suppressor Protein p53 by the Hsp70 and Hsp90 Chaperone Machineries. Mol Cell 74:816-830.e7. https://doi.org/10.1016/j.molcel.2019.03.026

63. Cheng F, McLaughlin PJ, Verderame MF, Zagon IS (2009) The OGF–OGFr Axis Utilizes the p16^INK4a^ and p21^WAF1/CIP1^ Pathways to Restrict Normal Cell Proliferation. Mol Biol Cell 20:319–327. https://doi.org/10.1091/mbc.e08-07-0681

64. Qi H, Grace Wright RH, Beato M, Price BD (2022) The ADP-ribose hydrolase NUDT5 is important for DNA repair. Cell Rep 41:111866. https://doi.org/10.1016/j.celrep.2022.111866

65. Hutten S, Kehlenbach RH (2006) Nup214 Is Required for CRM1-Dependent Nuclear Protein Export In Vivo. Mol Cell Biol 26:6772–6785. https://doi.org/10.1128/MCB.00342-06

66. Mukherjee PK, Funchain P, Retuerto M, Jurevic RJ, Fowler N, Burkey B, Eng C, Ghannoum MA (2017) Metabolomic analysis identifies differentially produced oral metabolites, including the oncometabolite 2-hydroxyglutarate, in patients with head and neck squamous cell carcinoma. BBA Clin 7:8–15. https://doi.org/10.1016/j.bbacli.2016.12.001

67. Zagon IS, Donahue RN, McLaughlin PJ (2009) Opioid growth factor-opioid growth factor receptor axis is a physiological determinant of cell proliferation in diverse human cancers. Am J Physiol-Regul Integr Comp Physiol 297:R1154–R1161. https://doi.org/10.1152/ajpregu.00414.2009

68. Kassovska-Bratinova S, Chen HS, Robert M-F, Perez-Cerdai C, Ugarte M, Chartrand C, Vobecky S, Kondo N, Mitchell’ GA (1996) Succinyl CoA: 3-Oxoacid CoA Transferase (SCOT): Human cDNA Cloning, Human Chromosomal Mapping to 5pl 3, and Mutation Detection in a SCOT-Deficient Patient. Am J Hum Genet

69. Ray Chaudhuri A, Nussenzweig A (2017) The multifaceted roles of PARP1 in DNA repair and chromatin remodelling. Nat Rev Mol Cell Biol 18:610–621. https://doi.org/10.1038/nrm.2017.53

70. Pommier Y, Leo E, Zhang H, Marchand C (2010) DNA topoisomerases and their poisoning by anticancer and antibacterial drugs. Chem Biol 17:421–433. https://doi.org/10.1016/j.chembiol.2010.04.012

71. Haider A, Wei Y-C, Lim K, Barbosa AD, Liu C-H, Weber U, Mlodzik M, Oras K, Collier S, Hussain MM, Dong L, Patel S, Alvarez-Guaita A, Saudek V, Jenkins BJ, Koulman A, Dymond MK, Hardie RC, Siniossoglou S, Savage DB (2018) PCYT1A Regulates Phosphatidylcholine Homeostasis from the Inner Nuclear Membrane in Response to Membrane Stored Curvature Elastic Stress. Dev Cell 45:481-495.e8. https://doi.org/10.1016/j.devcel.2018.04.012

72. Jardin F (2022) NFkB Pathway and Hodgkin Lymphoma. Biomedicines 10:2153. https://doi.org/10.3390/biomedicines10092153

73. Xu L, Hu J, Zhao Y, Hu J, Xiao J, Wang Y, Ma D, Chen Y (2012) PDCD5 interacts with p53 and functions as a positive regulator in the p53 pathway. Apoptosis 17:1235–1245. https://doi.org/10.1007/s10495-012-0754-x

74. Wang W, Song X-W, Zhao C-H (2016) Roles of programmed cell death protein 5 in inflammation and cancer (Review). Int J Oncol 49:1801–1806. https://doi.org/10.3892/ijo.2016.3706

75. Cuccaro A, Bellesi S, Galli E, Zangrilli I, Corrente F, Cupelli E, Fatone F, Maiolo E, Alma E, Viscovo M, D’Alò F, Annunziata S, Martini M, Rufini V, Giordano A, De Stefano V, Larocca LM, Hohaus S (2022) PD-L1 expression in peripheral blood granulocytes at diagnosis as prognostic factor in classical Hodgkin lymphoma. J Leukoc Biol 112:539–545. https://doi.org/10.1002/JLB.5AB0121-041R

76. Maki M, Takahara T, Shibata H (2016) Multifaceted Roles of ALG-2 in Ca(2+)-Regulated Membrane Trafficking. Int J Mol Sci 17:. https://doi.org/10.3390/ijms17091401

77. Vietri M, Radulovic M, Stenmark H (2020) The many functions of ESCRTs. Nat Rev Mol Cell Biol 21:25–42. https://doi.org/10.1038/s41580-019-0177-4

78. Qian B, Liu X, Gu X, Yang L, Chen D (2020) An in vitro study to explore the role of prolylcarboxypeptidase in non-small cell lung cancer. BIOCELL 44:19–26. https://doi.org/10.32604/biocell.2020.07859

79. Molitor L, Bacher S, Burczyk S, Niessing D (2021) The Molecular Function of PURA and Its Implications in Neurological Diseases. Front Genet 12:638217. https://doi.org/10.3389/fgene.2021.638217

80. Lai M-C (2003) A novel splicing regulator shares a nuclear import pathway with SR proteins. EMBO J 22:1359–1369. https://doi.org/10.1093/emboj/cdg126

81. Rutter J, Winge DR, Schiffman JD (2010) Succinate dehydrogenase – Assembly, regulation and role in human disease. Mitochondrion 10:393–401. https://doi.org/10.1016/j.mito.2010.03.001

82. Burgener SS, Leborgne NGF, Snipas SJ, Salvesen GS, Bird PI, Benarafa C (2019) Cathepsin G Inhibition by Serpinb1 and Serpinb6 Prevents Programmed Necrosis in Neutrophils and Monocytes and Reduces GSDMD-Driven Inflammation. Cell Rep 27:3646-3656.e5. https://doi.org/10.1016/j.celrep.2019.05.065

83. Boedtkjer E, Moreira JMA, Mele M, Vahl P, Wielenga VT, Christiansen PM, Jensen VED, Pedersen SF, Aalkjaer C (2013) Contribution of Na+,HCO3−-cotransport to cellular pH control in human breast cancer: A role for the breast cancer susceptibility locus NBCn1 (SLC4A7). Int J Cancer 132:1288–1299. https://doi.org/10.1002/ijc.27782

84. Axelsen TV, Olesen C, Khan D, Mohammadi A, Bouzinova EV, Nielsen CJF, Mele M, Hauerslev KR, Pedersen HL, Balling E, Vahl P, Tramm T, Christiansen PM, Boedtkjer E (2024) Antibodies toward Na+,HCO3–-cotransporter NBCn1/SLC4A7 block net acid extrusion and cause pH-dependent growth inhibition and apoptosis in breast cancer. Br J Cancer 130:1206–1220. https://doi.org/10.1038/s41416-024-02591-0

85. Zelko IN, Mariani TJ, Folz RJ (2002) Superoxide dismutase multigene family: a comparison of the CuZn-SOD (SOD1), Mn-SOD (SOD2), and EC-SOD (SOD3) gene structures, evolution, and expression. Free Radic Biol Med 33:337–349. https://doi.org/10.1016/s0891-5849(02)00905-x

86. Ju S, Singh MK, Han S, Ranbhise J, Ha J, Choe W, Yoon K-S, Yeo SG, Kim SS, Kang I (2024) Oxidative Stress and Cancer Therapy: Controlling Cancer Cells Using Reactive Oxygen Species. Int J Mol Sci 25:. https://doi.org/10.3390/ijms252212387

87. Bhattacharya K, Picard D (2021) The Hsp70–Hsp90 go-between Hop/Stip1/Sti1 is a proteostatic switch and may be a drug target in cancer and neurodegeneration. Cell Mol Life Sci 78:7257–7273. https://doi.org/10.1007/s00018-021-03962-z

88. Townsend PA, Scarabelli TM, Davidson SM, Knight RA, Latchman DS, Stephanou A (2004) STAT-1 Interacts with p53 to Enhance DNA Damage-induced Apoptosis. J Biol Chem 279:5811–5820. https://doi.org/10.1074/jbc.M302637200

89. Nair B, Menon A, Rithwik Kalidas M, Nath LR, Calina D, Sharifi-Rad J (2025) Modulating the JAK/STAT pathway with natural products: potential and challenges in cancer therapy. Discov Oncol 16:595. https://doi.org/10.1007/s12672-025-02369-7

90. Dong Y, Chen J, Chen Y, Liu S (2023) Targeting the STAT3 oncogenic pathway: Cancer immunotherapy and drug repurposing. Biomed Pharmacother 167:115513. https://doi.org/10.1016/j.biopha.2023.115513

91. Lin Y, Brown L, Hedley DW, Barber DL, Benchimol S (2002) The death-promoting activity of p53 can be inhibited by distinct signaling pathways. Blood 100:3990–4000. https://doi.org/10.1182/blood-2002-02-0504

92. Skinnider BF, Elia AJ, Gascoyne RD, Patterson B, Trumper L, Kapp U, Mak TW (2002) Signal transducer and activator of transcription 6 is frequently activated in Hodgkin and Reed-Sternberg cells of Hodgkin lymphoma. Blood 99:618–626. https://doi.org/10.1182/blood.V99.2.618

93. Carrozzo R, Verrigni D, Rasmussen M, De Coo R, Amartino H, Bianchi M, Buhas D, Mesli S, Naess K, Born AP, Woldseth B, Prontera P, Batbayli M, Ravn K, Joensen F, Cordelli DM, Santorelli FM, Tulinius M, Darin N, Duno M, Jouvencel P, Burlina A, Stangoni G, Bertini E, Redonnet‐Vernhet I, Wibrand F, Dionisi‐Vici C, Uusimaa J, Vieira P, Osorio AN, McFarland R, Taylor RW, Holme E, Ostergaard E (2016) Succinate‐CoA ligase deficiency due to mutations in *SUCLA2* and *SUCLG1* : phenotype and genotype correlations in 71 patients. J Inherit Metab Dis 39:243–252. https://doi.org/10.1007/s10545-015-9894-9

94. Zhang L, Liu R, Luan Y-Y, Yao Y-M (2018) Tumor Necrosis Factor-α Induced Protein 8: Pathophysiology, Clinical Significance, and Regulatory Mechanism. Int J Biol Sci 14:398–405. https://doi.org/10.7150/ijbs.23268

95. Horie R, Watanabe T (1998) CD30: expression and function in health and disease. Semin Immunol 10:457–470. https://doi.org/10.1006/smim.1998.0156

96. Shoshan-Barmatz V, Shteinfer-Kuzmine A, Verma A (2020) VDAC1 at the Intersection of Cell Metabolism, Apoptosis, and Diseases. Biomolecules 10:1485. https://doi.org/10.3390/biom10111485

97. Karunanithi Nivedita A, Shoshan-Barmatz V (2025) Etoposide-induced cancer cell death: roles of mitochondrial VDAC1 and calpain, and resistance mechanisms. Mol Oncol 19:1855–1875. https://doi.org/10.1002/1878-0261.13807

98. Raviv T, Shteinfer-Kuzmine A, Moyal MM, Shoshan-Barmatz V (2025) Resveratrol’s Pro-Apoptotic Effects in Cancer Are Mediated Through the Interaction and Oligomerization of the Mitochondrial VDAC1. Int J Mol Sci 26:3963. https://doi.org/10.3390/ijms26093963

99. Varughese JT, Buchanan SK, Pitt AS (2021) The Role of Voltage-Dependent Anion Channel in Mitochondrial Dysfunction and Human Disease. Cells 10:. https://doi.org/10.3390/cells10071737
